# G-computation, propensity score-based methods, and targeted maximum likelihood estimator for causal inference with different covariates sets: a comparative simulation study

**DOI:** 10.1038/s41598-020-65917-x

**Published:** 2020-06-08

**Authors:** Arthur Chatton, Florent Le Borgne, Clémence Leyrat, Florence Gillaizeau, Chloé Rousseau, Laetitia Barbin, David Laplaud, Maxime Léger, Bruno Giraudeau, Yohann Foucher

**Affiliations:** 1 INSERM UMR 1246 - SPHERE, Université de Nantes, Université de Tours, Nantes, France; 2 A2COM-IDBC, Pacé, France; 3 Department of Medical Statistics & Cancer Survival Group, London School of Hygiene and Tropical Medicine, London, UK; 4 Centre Hospitalier Universitaire de Nantes, Nantes, France; 5 INSERM CIC1414, CHU Rennes, Rennes, France; 6 Centre de Recherche en Transplantation et Immunologie INSERM UMR1064, Université de Nantes, Nantes, France; 7 Département d’Anesthésie-Réanimation, Centre Hospitalier Universitaire d’Angers, Angers, France; 8 INSERM CIC1415, CHRU de Tours, Tours, France

**Keywords:** Epidemiology, Epidemiology, Risk factors

## Abstract

Controlling for confounding bias is crucial in causal inference. Distinct methods are currently employed to mitigate the effects of confounding bias. Each requires the introduction of a set of covariates, which remains difficult to choose, especially regarding the different methods. We conduct a simulation study to compare the relative performance results obtained by using four different sets of covariates (those causing the outcome, those causing the treatment allocation, those causing both the outcome and the treatment allocation, and all the covariates) and four methods: g-computation, inverse probability of treatment weighting, full matching and targeted maximum likelihood estimator. Our simulations are in the context of a binary treatment, a binary outcome and baseline confounders. The simulations suggest that considering all the covariates causing the outcome led to the lowest bias and variance, particularly for g-computation. The consideration of all the covariates did not decrease the bias but significantly reduced the power. We apply these methods to two real-world examples that have clinical relevance, thereby illustrating the real-world importance of using these methods. We propose an R package *RISCA* to encourage the use of g-computation in causal inference.

## Introduction

The randomised controlled trial (RCT) remains the primary design for evaluating the marginal (population average) causal effect of a treatment, *i*.*e*., the average treatment effect between two hypothetical worlds where: i) everyone is treated and ii) everyone is untreated^1^. Indeed, a well-designed RCT with a sufficient sample size ensures the baseline comparability between groups, thus allowing the estimation of a marginal causal effect. Nevertheless, it is well established that RCT is performed under optimal circumstances (*e*.*g*., over-representation of treatment-adherent patients, low frequency of morbidity), which may be different from real-life practices^2^. Observational studies have the advantage of limiting the issue of external validity, but treated and untreated patients are often non-comparable, leading to a high risk of confounding bias.

To reduce such confounding bias, the vast majority of observational studies have been based on multivariable models (mainly linear, logistic, or Cox models), allowing for the direct estimation of conditional (subject-specific) effects, *i*.*e*., the average effect across sub-populations of subjects who share the same characteristics. Several methods have been proposed to estimate marginal causal effects in observational studies, amongst which propensity score (PS)-based methods are increasingly used in epidemiology and medical research^3^.

Propensity score-based methods make use of the PS in four different ways to account for confounding, namely matching, stratification, conditional adjustment^4^ and inverse probability of treatment weighting (IPTW)^5^. Stratification and conditional adjustment on PS are associated with the highest bias^6–8^, because the two methods estimate the conditional treatment effect rather than the marginal causal effect. Matching on PS remains the most common approach with a usage rate of 83.8% in 303 surgical studies using PS-based methods^9^ and 68.9% in 296 medical studies (without restriction regarding the field) also using PS-methods^10^. The IPTW appears to be less biased and associated with a lower variance than matching in several studies^8,11–14^. Nevertheless, in particular settings, full matching (FM) was associated with lower mean square error (MSE) in other studies^15–17^.

Multivariable models, even non-linear ones, can also be used to indirectly estimate the marginal causal effect with g-computation (GC)^18^. This method is also called the parametric g-formula^1^ or (g-)standardisation^19^ in the literature. Snowden *et al*.^20^ and Wang *et al*.^21^ detailed the corresponding methodology for estimating the average treatment (*i*.*e*., marginal causal) effect on the entire population (ATE) or only on the treated (ATT), respectively. The ATE is the average effect, at the population level, of moving an entire population from untreated to treated. The ATT is the average effect of treatment on those subjects who ultimately received the treatment^22^. Furthermore, some authors^23,24^ have proposed combinations of GC and PS to improve the estimation of the marginal causal effect. These methods are known as doubly robust estimators (DRE) because they require the specification of both the outcome (for GC) and treatment allocation (for PS) mechanisms to minimise the impact of model misspecification. Indeed, these estimators are consistent as long as either the outcome model or the treatment model is estimated correctly^25^.

Each of these methods carries out the adjustment in different ways, but all of these methods rely on the same condition: a correct specification of the PS or the outcome model^1^. In practice, a common issue is choosing the set of covariates to include to obtain the best performance in terms of bias and precision. Three simulation studies^7,26,27^ have investigated this issue for PS-based methods. They studied four sets of covariates: those causing the outcome, those causing the treatment allocation, those are a common cause of both the treatment allocation and the outcome, and all the covariates. For the rest of this paper, we called these strategies the *outcome set*, the *treatment set*, the *common set* and the *entire set*, respectively. These studies argued in favour of the outcome or common sets for PS-based methods, but it is not immediately clear that such works will generalise to other methods of causal inference. Brookhart *et al*.^26^ and Lefebvre *et al*.^27^ focused on count and continuous outcomes. Austin *et al*.^7^ investigated binary outcomes on matching, stratification and adjustment on PS. However, GC and DRE also require the correct specification of the outcome model with a potentially different set of covariates. Recent works have shown that efficiency losses can accompany the inclusion of unnecessary covariates^28–31^. De Luna *et al*.^32^ also highlighted the variance inflation caused by the treatment set. In contrast, VanderWeele and Shpitser^33^ suggested the inclusion of both the outcome and the treatment sets.

Before selecting the set of covariates, one needs to select the method to employ. Several studies have compared the performances of GC, PS-based methods and DRE in a point treatment study to estimate the ATE^13,23,25,34–36^. Half of these studies investigated a binary outcome^13,25,34^. Only Colson *et al*.^17^ studied the ATT, but they focused on a continuous outcome. Except in Neugebauer and van der Laan^25^, these studies only investigated the ATE (or ATT) defined as a risk difference. The CONSORT recommended the presentation of both the absolute and the relative effect sizes for a binary outcome, “*as neither the relative measure nor the absolute measure alone gives a complete picture of the effect and its implications*”^37^. None of these studies was interested in the set of covariates necessary to obtain the best performance.

In our study, we sought to compare different sets of covariates to consider to estimate a marginal causal effect. Moreover, we compared GC, PS-based methods and DRE for both the ATE and ATT, either in terms of risk difference or marginal causal OR. Three main types of outcome are used in epidemiology and medical research: continuous, binary and time-to-event outcomes. We focused on a binary outcome because i) a continuous outcome is often appealing for linear regression where the two conditional and marginal causal effects are collapsible^38^, and ii) time-to-event analyses present additional methodological difficulties, such as the time-dependant covariate distribution^39^. We also limit our study to a binary treatment, as in the current literature, and the extension to three or more modalities is beyond the scope of our study.

The paper is structured as follows. In the next section, the methods are detailed. The third section presents the design and results of the simulations. In the fourth section, we consider two real data sets. Finally, we discuss our results in the last section.

## Methods

### Setting and notations

Let $A$ denote the binary treatment of interest ($A=1$ for treated patients and $0$ otherwise), $Y$ denote the binary outcome ($Y=1$ for events and $0$ otherwise), and $L$ denote a set of baseline covariates. Consider a sample of size $n$ in which one can observe the realisations of these random variables: $a$, $y$, and $l$, respectively. Define ${\pi }_{a}=E(P(Y=1|do(A=a),L))$ or ${\pi }_{a}=E(P(Y=1|do(A=a),L)|A=1)$ as the expected proportions of event if the entire (ATE) or the treated (ATT) populations were treated ($do(A=1)$) or untreated ($do(A=0)$), respectively^40^. From these probabilities, the risk difference can be estimated as $\Delta \pi ={\pi }_{1}-{\pi }_{0}$ and the log of the marginal causal OR estimated as $\theta ={\rm{logit}}({\pi }_{1})/{\rm{logit}}({\pi }_{0})$, where logit(•) = log(•/(1 − •)). The methods described bellow allow for the estimation of both the ATE and the ATT effects.

Causal inference requires the three following assumptions, called *identifiability conditions*: i) The values of exposure under comparisons correspond to well-defined interventions that, in turn, correspond to the versions of treatment in the data. ii) The conditional probability of receiving every value of treatment, though not decided by the investigators, depends only on the measured covariates. iii) The conditional probability of receiving every value of the treatment is greater than zero, *i*.*e*., is positive. These assumptions are known as *consistency*, *(conditional) exchangeability* and *positivity*, respectively^1^. However, PS-based methods rely on treatment allocation modelling to obtain a pseudo-population in which the confounders are balanced across treatment groups. Covariate balance can be checked by computing the standardised difference of the covariates included in the PS between the two treatment groups^10^. In contrast, GC relies on outcome modelling to predict hypothetical outcomes for each subject under each treatment regimen. Note that one can ignore the lack of positivity if one is willing to rely on Q-model extrapolation^1^. As is the case for standard regression models, these methods also require the assumptions of no interference, no measurement error and no model misspecification.

### Weighting on the inverse of the propensity score

Formally, the PS is ${p}_{i}=P({A}_{i}=1|{L}_{i})$, *i*.*e*. the probability that subject $i$ ($i=1,\ldots ,n$) will be treated according to his or her characteristics ${L}_{i}$ at the time of the treatment allocation^4^. It is often estimated using a logistic regression. The IPTW makes it possible to reduce confounding by correcting the contribution of each subject $i$ by a weight ${\omega }_{i}$. For ATE, Xu *et al*.^41^ defined ${\omega }_{i}={A}_{i}P({A}_{i}=1)/{p}_{i}+(1-{A}_{i})P({A}_{i}=0)/(1-{p}_{i})$. The use of stabilised weights has been shown to produce a suitable estimate of the variance even when there are subjects with extremely large weights^5,41^. For ATT, Morgan and Todd^42^ defined ${\omega }_{i}={A}_{i}+(1-{A}_{i}){p}_{i}/(1-{p}_{i})$. Based on ${\omega }_{i}$, the following weighted univariate logistic regression can be fitted: ${\rm{logit}}\{P(Y=1|A)\}={\hat{\alpha }}_{0}+{\hat{\alpha }}_{1}A$, resulting in ${\hat{\pi }}_{0}={(1+\exp (-{\hat{\alpha }}_{0}))}^{-1}$, ${\hat{\pi }}_{1}={(1+\exp (-{\hat{\alpha }}_{0}-{\hat{\alpha }}_{1}))}^{-1}$, and $\hat{\theta }={\hat{\alpha }}_{1}$. To obtain $\widehat{var}(\hat{\theta })$, we used a robust sandwich-type variance estimator^5^ with the R package *sandwich*^43^.

### Full Matching on the propensity score

The FM minimises the average within-stratum differences in the PS between treated and untreated subjects^16^. Then, two weighting systems can be applied in each stratum, making it possible to estimate either the ATE or the ATT unlike other matching methods which can only estimate the ATT^44^. If $t$ and $u$ denote the number of treated and untreated subjects in a given stratum, one can define the weight for a subject $i$ in this stratum as ${\omega }_{i}={A}_{i}P(A=1)(t+u)/u+(1-{A}_{i})(1-P(A=1))(t+u)/t$ for ATE and ${\omega }_{i}={A}_{i}+(1-{A}_{i})t/u$ for ATT^16^. In the latter case, the weights of untreated subjects are rescaled such that the sum of the untreated weights across all the matched sets is equal to the number of untreated subjects: ${\tilde{\omega }}_{i}={\omega }_{i}\times {\sum }_{j=1}^{n}(1-{A}_{j})/{\sum }_{j=1}^{n}{\omega }_{j}(1-{A}_{j})$^45^. From the resulting paired data set, we fitted a weighted univariate logistic regression, and the rest of the data analysis is tantamount to IPTW. We used the R package *MatchIt*^45^ to generate the pairs.

### G-computation

Consider the following multivariable logistic regression ${\rm{logit}}\{P(Y=1|A,L)\}=\gamma A+\beta L$. This regression is frequently called the *Q-model*^20^. Once fitted, one can compute for all subjects $\hat{P}({Y}_{i}=1|do({A}_{i}=1),{L}_{i})$ and $\hat{P}({Y}_{i}=1|do({A}_{i}=0),{L}_{i})$, *i*.*e*. the two expected probabilities of events if they were treated or untreated^20^. For ATE, one can then obtain ${\hat{\pi }}_{a}={n}^{-1}{\sum }_{i}\hat{P}({Y}_{i}=1|do({A}_{i}=a),{L}_{i})$. The same procedure can be performed amongst the treated patients for ATT^21^. For implementation in practice, consider a treated subject (${A}_{i}=1$) included in the fit of the Q-model. Thanks to this model, one can then compute for this subject his or her predicted probabilities of the event if he or she received the treatment ($do({A}_{i}=1)$) or not ($do({A}_{i}=0)$). Computing these predicted probabilities for all the subjects, one can obtain two vectors of probabilities if the entire sample were treated or not. The corresponding means correspond to ${\hat{\pi }}_{1}$ and ${\hat{\pi }}_{0}$, respectively. We obtained $\widehat{var}(\hat{\theta })$ by simulating the parameters of the multivariable logistic regression assuming a multinormal distribution^46^. Note that we could have used bootstrap resampling instead. However, regarding the computational burden of bootstrapping and the similar results obtained by Aalen *et al*.^46^, the variance estimates in the simulation study were only based on parametric simulations. We used both bootstrap resampling and parametric simulations in the applications.

### Targeted Maximum Likelihood Estimator

Amongst the several existing DREs, we focused on the targeted maximum likelihood estimator (TMLE)^24^, for which estimators of ATE and ATT have been proposed^47^. The TMLE begins by fitting the Q-model to estimate the two expected hypothetical probabilities of events ${\hat{\pi }}_{1}$ and ${\hat{\pi }}_{0}$. An additional “targeting” step involves estimation of the treatment allocation mechanism, *i*.*e*., the PS $P({A}_{i}=1|{L}_{i})$, which is then used to update the initial estimates obtained by GC. In the presence of residual confounding, the PS provides additional information to improve the initial estimates. Finally, the updated estimates of ${\hat{\pi }}_{1}$ and ${\hat{\pi }}_{0}$ are used to generate $\widehat{\Delta \pi }$ or $\hat{\theta }$. We used the efficient influence curve to obtain standard errors^47,48^. A recent tutorial provides a step-by-step guided implementation of TMLE^49^.

## Simulation study

### Design

**Figure 1:**
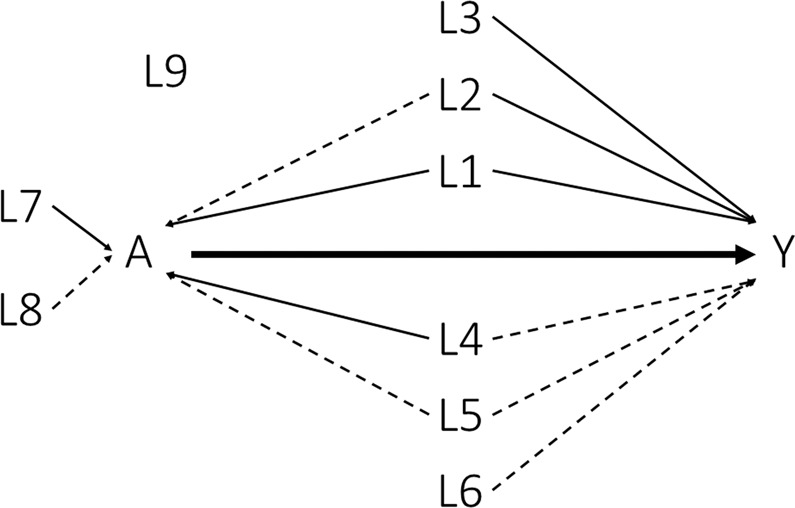
Causal diagram. Solid lines corresponded to a strong association (OR = 6.0) and dashed lines to a moderate one (OR = 1.5).

We used a close data generating procedure from previous studies on PS models^7,50^. We generated the data in three steps. i) Nine covariates (*L*_1_, …, *L*_9_) were independently simulated from a Bernoulli distribution with a parameter equal to 0.5 for all covariates. ii) We generated the treatment $A$ according to a Bernoulli distribution with a probability obtained by the logistic model with the following linear predictor: ${\gamma }_{0}+{\gamma }_{1}{L}_{1}+\cdots +{\gamma }_{9}{L}_{9}$. We fixed the parameter ${\gamma }_{0}$ at −3.3 or −5.2 to obtain a percentage of treated patients equal to 50% for scenarios related to ATE and 20% for ATT, respectively. iii) We simulated the event $Y$ using a Bernoulli distribution with a probability obtained by the logistic model with the following linear predictor: ${\beta }_{0}+{\beta }_{1}A+{\beta }_{2}{L}_{1}+\cdots +{\beta }_{10}{L}_{9}$. We set the parameter ${\beta }_{1}$ for a conditional OR at 0 (the null hypothesis is no treatment effect) or 2 (the alternative hypothesis is a negative impact of treatment). We also fixed the parameter ${\beta }_{0}$ at −3.65 and −3.5 to obtain a percentage of the event close to 50% in ATE and ATT, respectively. Figure 1 presents the values of the regression coefficients ${\gamma }_{1}$ to ${\gamma }_{9}$ and ${\beta }_{1}$ to ${\beta }_{10}$. We considered four covariates sets as explained in the introduction: the outcome set included the covariates ${L}_{1}$ to ${L}_{6}$, the treatment set included the covariates ${L}_{1},{L}_{2},{L}_{4},{L}_{5},{L}_{7},{L}_{8}$, the common set included the covariates ${L}_{1},{L}_{2},{L}_{4},{L}_{5}$, and the entire set included the covariates ${L}_{1}$ to ${L}_{9}$. For each of the four methods and the four covariate sets, we studied the performance under different sample sizes: $n\,=$ 100, 300, 500 and 2000. For each scenario, we randomly generated 10 000 data sets. We computed the theoretical values of ${\pi }_{1}$ and ${\pi }_{0}$ by averaging the values of ${\pi }_{1}$ and ${\pi }_{0}$ obtained from univariate logistic models (treatment as the only covariate) fitted from data sets simulated as above, except that the treatment $A$ was simulated independently of the covariates $L$^50^. We reported the following criteria: i) the percentage of non-convergence, ii) the mean absolute bias (*e*.*g*., $E(\hat{\theta })-\theta$), iii) the MSE ($E[(\hat{\theta }-\theta {)}^{2}]$), the variance estimation bias $({\rm{VEB}}=100\times (\sqrt{E[\widehat{V}ar(\hat{\theta })]}/\sqrt{Var(\hat{\theta })}-1))$^51^, the empirical coverage rate of the nominal 95% confidence intervals (CIs), defined as the percentage of 95% CI including the theoretical value, the type I error, defined as the percentage of rejection of the null hypothesis under the null hypothesis, and the statistical power, defined as the percentage of rejections of the null hypothesis under the alternative hypothesis. The MSE was our primary performance measure of interest because it combines bias and variance. We assumed that the identifiability conditions hold in these scenarios. We further performed the same simulations by omitting ${L}_{1}$ in the PS or in the Q-model to evaluate the impact of an unmeasured confounder. We performed all the analyses using R version 3.6.0^52^.

## Results

### Convergence

**Figure 2:**
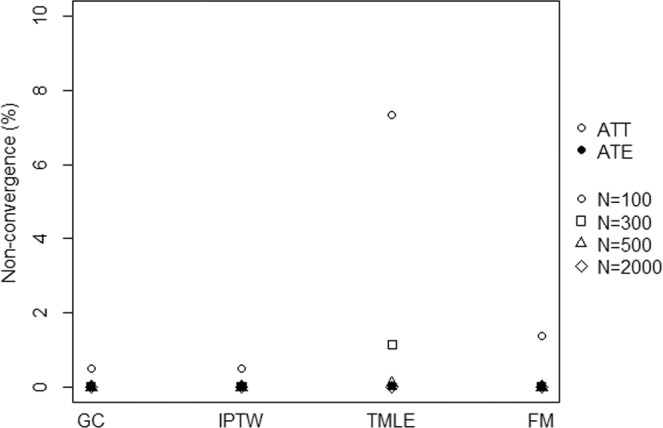
Percentage of simulation iterations which did not converge according to the methods.

Non-convergence only occurred for ATT estimation when sample sizes were lower or equal to 300 subjects (see Fig. 2). The GC, IPTW and FM had a minimal convergence percentage higher than 98%, even under small sample size (n = 100). Similarly, TMLE experienced some difficulty in converging for ATT estimation in the medium-sized sample (n = 300). However, they experienced severe difficulty in converging in the small sample with a convergence percentage of approximately 92%.

### Mean bias

As expected with the common set, the mean absolute bias of $\theta$ was close to zero for GC, IPTW and TMLE when the three identifiability assumptions hold with a maximum at −0.028 given moderate sample size (n = 300) under the alternative hypothesis for ATT estimation (Table 1). Note that the three other covariate sets led to a bias close to zero with a maximum of 0.053 for TMLE with the entire set given small sample size (n = 100) under the alternative hypothesis for ATE estimation (Table 2). Furthermore, FM was also associated with a similar bias with a maximum of 0.082 given a small sample size (n = 100), with the treatment set under the alternative hypothesis for the ATE estimation. With an unmeasured confounder, the bias increased in all scenarios with a minimum of 0.456 for GC with the common set given a large sample size for the ATT estimation (see Online Supporting Information (OSI) for complete results). The results were similar under the null hypothesis (see OSI).

**Table 1:** Simulation results comparing the ATT estimation under the alternative hypothesis.

n	method	selection strategy	mean bias				log OR				
			*π*_0_	*π*_1_	Δ*π*	log OR	MSE	MSE*	VEB (%)	coverage (%)	power (%)
100	GC	outcome	0.000	−0.001	−0.001	0.012	0.526	0.716	−6.2	94.1	17.7
		treatment	0.002	−0.001	−0.003	0.006	0.580	0.786	−5.7	94.1	14.0
		common	0.002	−0.001	−0.003	0.006	0.552	0.735	−4.2	94.8	15.1
		entire	−0.001	−0.001	−0.001	0.013	0.558	0.768	−8.8	93.3	16.9
	IPTW	outcome	0.000	−0.001	−0.001	0.008	0.578	0.727	10.8	97.3	7.8
		treatment	−0.000	−0.001	−0.001	0.000	0.716	0.837	−1.2	95.1	9.8
		common	0.002	−0.001	−0.003	0.003	0.587	0.743	6.6	96.8	8.8
		entire	−0.003	−0.001	0.002	0.005	0.741	0.838	−1.5	95.2	9.6
	TMLE	outcome	−0.001	−0.001	0.000	0.002	0.694	0.794	30.0	95.7	5.8
		treatment	0.000	−0.001	−0.001	−0.020	0.876	0.955	183.3	98.8	1.0
		common	−0.000	−0.001	−0.001	−0.001	0.702	0.794	10.4	95.3	7.3
		entire	−0.003	−0.001	0.001	−0.013	0.886	0.953	412.2	98.8	0.5
	FM	outcome	−0.004	−0.001	0.003	0.022	0.665	0.787	−16.7	90.1	18.9
		treatment	−0.006	−0.001	0.004	0.017	0.822	0.911	−32.3	81.3	25.2
		common	−0.001	−0.001	−0.000	0.010	0.653	0.795	−15.3	91.0	17.5
		entire	−0.008	−0.001	0.006	0.022	0.842	0.921	−33.8	80.3	26.7
300	GC	outcome	0.001	−0.001	−0.002	−0.021	0.283	0.555	−1.6	94.5	43.6
		treatment	0.002	−0.001	−0.003	−0.024	0.319	0.606	−2.3	94.3	35.2
		common	0.002	−0.001	−0.003	−0.023	0.304	0.561	−1.5	94.8	38.5
		entire	0.001	−0.001	−0.002	−0.022	0.297	0.600	−2.6	94.0	39.9
	IPTW	outcome	0.002	−0.001	−0.003	−0.027	0.301	0.556	16.4	97.9	24.0
		treatment	0.001	−0.001	−0.002	−0.026	0.372	0.628	6.6	96.2	21.4
		common	0.003	−0.001	−0.004	−0.028	0.318	0.563	9.1	96.8	26.1
		entire	0.001	−0.001	−0.002	−0.025	0.361	0.622	11.7	97.2	20.0
	TMLE	outcome	0.000	−0.001	−0.001	−0.023	0.358	0.577	−2.3	93.6	29.0
		treatment	0.002	−0.001	−0.003	−0.035	0.454	0.683	51.2	99.1	6.8
		common	0.001	−0.001	−0.002	−0.023	0.378	0.582	−3.5	93.0	26.5
		entire	0.002	−0.001	−0.003	−0.035	0.432	0.674	81.8	99.3	4.4
	FM	outcome	−0.000	−0.001	−0.001	−0.020	0.351	0.579	−11.7	91.9	37.2
		treatment	−0.001	−0.001	−0.000	−0.022	0.444	0.656	−30.2	82.7	38.9
		common	0.001	−0.001	−0.002	−0.024	0.363	0.587	−14.6	90.4	36.9
		entire	−0.001	−0.001	0.000	−0.020	0.439	0.662	−29.3	83.2	39.1
500	GC	outcome	0.001	−0.001	−0.002	−0.014	0.217	0.509	−1.1	94.7	64.5
		treatment	0.001	−0.001	−0.002	−0.014	0.245	0.556	−1.5	94.4	53.6
		common	0.001	−0.001	−0.002	−0.015	0.233	0.618	−0.8	94.8	57.6
		entire	0.001	−0.001	−0.002	−0.014	0.228	0.552	−2.0	94.2	60.5
	IPTW	outcome	0.002	−0.001	−0.003	−0.019	0.230	0.509	16.5	97.9	43.3
		treatment	0.000	−0.001	−0.001	−0.013	0.285	0.574	6.8	96.6	35.4
		common	0.002	−0.001	−0.003	−0.018	0.244	0.514	9.2	96.8	43.7
		entire	0.000	−0.001	−0.001	−0.014	0.274	0.571	12.3	97.2	33.9
	TMLE	outcome	0.001	−0.001	−0.002	−0.015	0.272	0.521	−4.7	93.4	48.5
		treatment	0.001	−0.001	−0.002	−0.018	0.347	0.618	35.0	99.1	15.9
		common	0.000	−0.001	−0.001	−0.013	0.289	0.527	−4.8	93.1	43.7
		entire	0.001	−0.001	−0.002	−0.019	0.328	0.611	51.1	99.3	12.9
	FM	outcome	0.001	−0.001	−0.002	−0.015	0.265	0.525	−9.9	92.4	53.0
		treatment	−0.001	−0.001	−0.000	−0.011	0.346	0.597	−31.0	82.7	51.7
		common	0.001	−0.001	−0.001	−0.014	0.283	0.530	−15.8	90.1	52.3
		entire	−0.002	−0.001	0.001	−0.008	0.340	0.596	−29.8	83.2	52.6
2000	GC	outcome	0.000	0.000	−0.000	−0.002	0.108	0.479	−1.7	94.7	99.6
		treatment	0.001	0.000	−0.000	−0.003	0.122	0.524	−1.2	94.8	98.6
		common	0.001	0.000	−0.000	−0.003	0.116	0.480	−0.9	94.7	99.1
		entire	0.000	0.000	−0.000	−0.002	0.113	0.523	−1.8	94.5	99.4
	IPTW	outcome	0.002	0.000	−0.001	−0.006	0.113	0.478	16.3	97.6	98.1
		treatment	0.000	0.000	−0.000	−0.002	0.138	0.539	7.9	96.4	93.0
		common	0.002	0.000	−0.001	−0.006	0.120	0.480	9.4	97.0	97.7
		entire	0.000	0.000	−0.000	−0.002	0.131	0.537	13.9	97.4	93.6
2000	TMLE	outcome	0.001	0.000	−0.000	−0.002	0.132	0.483	−5.9	93.3	97.5
		treatment	0.000	0.000	0.000	−0.002	0.169	0.568	18.2	98.2	71.8
		common	−0.000	0.000	0.000	−0.000	0.142	0.486	−5.6	93.6	95.5
		entire	0.001	0.000	−0.000	−0.004	0.158	0.565	23.5	98.6	75.3
	FM	outcome	0.000	0.000	−0.000	−0.002	0.134	0.484	−12.0	91.6	97.7
		treatment	0.001	0.000	−0.000	−0.005	0.203	0.548	−41.6	74.6	89.9
		common	0.001	0.000	−0.000	−0.003	0.149	0.485	−20.5	88.5	96.7
		entire	0.000	0.000	0.000	−0.002	0.162	0.543	−26.9	84.5	94.8

*MSE in the presence of an unmeasured confounder. Theoretical values: ${\pi }_{1}=0.701,{\pi }_{0}=0.589,\theta =0.492$.

**Table 2:** Simulation results comparing the ATE estimation under the alternative hypothesis.

n	method	set	mean bias				log OR				
			*π*_0_	*π*_1_	Δ*π*	log OR	MSE	MSE*	VEB (%)	coverage (%)	power (%)
100	GC	outcome	−0.001	−0.002	−0.001	−0.003	0.404	0.634	−7.3	93.2	24.7
		treatment	−0.002	−0.001	0.000	0.004	0.477	0.727	−9.5	92.4	19.9
		common	−0.001	−0.002	−0.001	−0.002	0.434	0.650	−6.6	93.5	22.1
		entire	−0.002	−0.001	0.001	0.003	0.450	0.714	−11.4	91.8	22.6
	IPTW	outcome	−0.003	−0.001	0.001	0.011	0.464	0.646	12.1	97.4	12.1
		treatment	−0.006	0.002	0.008	0.046	0.633	0.769	−7.6	93.8	16.7
		common	−0.002	−0.001	0.001	0.010	0.480	0.657	6.3	96.3	13.5
		entire	−0.006	0.003	0.009	0.053	0.647	0.773	−7.2	94.7	16.4
	TMLE	outcome	−0.001	−0.002	−0.000	0.003	0.438	0.642	−14.3	89.5	26.9
		treatment	−0.004	0.002	0.006	0.039	0.572	0.757	−24.9	84.3	27.5
		common	−0.001	−0.002	−0.001	0.002	0.469	0.657	−10.7	90.9	21.2
		entire	−0.005	0.003	0.007	0.043	0.544	0.748	−30.7	80.9	34.3
	FM	outcome	−0.005	0.002	0.006	0.039	0.549	0.710	−24.3	87.1	28.5
		treatment	−0.009	0.005	0.014	0.082	0.677	0.832	−37.7	78.0	35.1
		common	−0.005	0.001	0.006	0.038	0.563	0.713	−26.3	85.8	29.1
		entire	−0.007	0.006	0.014	0.082	0.674	0.830	−37.3	78.1	34.8
300	GC	outcome	−0.000	−0.000	0.000	0.001	0.221	0.532	−1.9	94.5	59.8
		treatment	−0.000	−0.000	0.000	0.001	0.259	0.608	−2.8	94.3	47.4
		common	−0.000	−0.000	0.000	0.001	0.237	0.539	−1.2	94.8	53.5
		entire	−0.000	−0.000	0.000	0.001	0.241	0.600	−3.4	94.0	53.0
	IPTW	outcome	−0.001	−0.000	0.001	0.006	0.239	0.533	20.2	98.0	34.7
		treatment	−0.002	0.000	0.003	0.014	0.330	0.615	4.6	96.0	29.5
		common	−0.001	−0.000	0.001	0.006	0.252	0.541	13.3	97.4	36.5
		entire	−0.002	0.000	0.002	0.013	0.326	0.607	7.9	96.6	28.5
	TMLE	outcome	−0.000	−0.001	−0.000	0.000	0.233	0.532	−3.0	93.9	54.2
		treatment	−0.001	0.000	0.002	0.009	0.310	0.612	−10.4	90.6	40.2
		common	−0.001	−0.001	0.000	0.001	0.249	0.540	−1.5	94.6	48.1
		entire	−0.001	0.000	0.001	0.008	0.290	0.603	−13.2	89.6	46.1
	FM	outcome	−0.002	0.000	0.002	0.010	0.294	0.552	−20.2	88.7	51.6
		treatment	−0.003	0.003	0.006	0.032	0.389	0.652	−39.3	77.0	53.3
		common	−0.001	−0.000	0.001	0.008	0.315	0.588	−25.5	86.2	51.3
		entire	−0.003	0.003	0.006	0.032	0.377	0.644	−37.4	77.8	52.2
500	GC	outcome	−0.000	0.000	0.001	0.003	0.168	0.501	−0.4	94.8	81.1
		treatment	−0.000	0.000	0.001	0.002	0.198	0.573	−1.0	94.8	69.0
		common	−0.000	0.000	0.000	0.002	0.183	0.505	−0.7	94.9	75.0
		entire	−0.000	0.000	0.001	0.004	0.183	0.569	−1.0	94.8	75.3
	IPTW	outcome	−0.001	0.000	0.001	0.005	0.180	0.501	22.2	98.3	58.5
		treatment	−0.001	0.001	0.001	0.007	0.248	0.573	8.1	96.5	42.3
		common	−0.001	0.000	0.001	0.005	0.193	0.505	13.8	97.3	58.6
		entire	−0.001	0.000	0.001	0.006	0.239	0.569	13.1	97.2	41.3
	TMLE	outcome	−0.000	0.000	0.000	0.002	0.177	0.501	−0.8	94.7	76.8
		treatment	−0.000	0.000	0.000	0.003	0.234	0.571	−5.9	92.7	56.1
		common	−0.000	0.000	0.000	0.002	0.190	0.505	−0.5	94.7	69.7
		entire	−0.000	0.000	0.000	0.003	0.218	0.566	−7.5	91.8	63.1
	FM	outcome	−0.001	0.000	0.001	0.005	0.219	0.518	−17.5	89.8	70.1
		treatment	−0.002	0.002	0.003	0.018	0.302	0.598	−39.8	76.2	65.5
		common	−0.001	−0.000	0.001	0.005	0.266	0.555	−31.8	82.3	66.4
		entire	−0.002	0.002	0.004	0.019	0.289	0.592	−37.1	78.3	66.2
2000	GC	outcome	−0.000	−0.000	−0.000	−0.001	0.085	0.482	−0.6	94.6	100.0
		treatment	0.000	−0.001	−0.001	−0.003	0.099	0.550	−0.6	94.7	99.8
		common	0.000	−0.001	−0.001	−0.003	0.092	0.483	−0.8	94.7	99.9
		entire	−0.000	−0.000	−0.000	−0.001	0.091	0.550	−0.6	94.7	99.9
	IPTW	outcome	−0.000	−0.000	0.000	0.002	0.090	0.482	21.2	98.2	99.8
		treatment	0.000	−0.001	−0.001	−0.002	0.122	0.547	9.3	96.7	95.1
		common	−0.000	−0.000	0.000	0.001	0.096	0.483	13.5	97.3	99.7
		entire	0.000	−0.000	−0.001	−0.002	0.117	0.546	14.3	97.5	95.6
2000	TMLE	outcome	−0.000	−0.000	−0.000	−0.001	0.088	0.482	−0.6	94.8	100.0
		treatment	0.000	−0.001	−0.001	−0.003	0.116	0.545	−2.2	94.4	98.7
		common	0.000	−0.000	−0.001	−0.002	0.095	0.483	−0.3	94.8	99.9
		entire	0.000	−0.000	−0.001	−0.002	0.108	0.544	−2.6	94.1	99.4
	FM	outcome	−0.000	−0.000	−0.000	0.000	0.129	0.497	−29.9	82.9	99.0
		treatment	−0.001	−0.000	0.000	0.003	0.169	0.569	−46.6	70.6	96.2
		common	0.000	−0.000	−0.001	−0.001	0.205	0.534	−55.9	61.1	92.7
		entire	−0.000	−0.000	0.000	0.002	0.145	0.549	−37.7	77.9	98.2

*MSE in the presence of an unmeasured confounder. Theoretical values: ${\pi }_{1}=0.557,{\pi }_{0}=0.441,\theta =0.466$.

### Variance

For all methods, the outcome set led to the lowest MSE, followed closely by the common set. G-computation led to the lowest MSE and FM to the highest. In ATT, IPTW had lower MSE than TMLE. Note that the VEB was particularly high for FM in all ATE scenarios with a minimum of −17.5% (n = 500 with the outcome set). For the ATT, FM also had a higher VEB than other methods, apart from TMLE with the treatment or entire sets in sample sizes of fewer than 2000 subjects. In the presence of an unmeasured confounder, the MSE increased in all scenarios in agreement with the increase in bias. The VEBs did not change notably with an unmeasured confounder.

### Coverage and error rates

G-computation produced coverage rates close to 95%, except for ATE in a small sample size leading to an anti-conservative 95% CIs with a minimum of 91.7% with the entire set under the null hypothesis. Anti-conservatives 95% CIs were also produced by FM in all scenarios, and by TMLE given a small sample size. Conversely, conservative 95% CIs were obtained when using TMLE for the ATT with the entire or the treatment sets, and when using IPTW for ATT or ATE with the outcome or the common sets.

Lending confidence to these results, the type I error was close to 5% for GC in all scenarios and may vary for other methods. The power was more impacted by the choice of the covariate set. The outcome set led to the highest power for GC.

## Applications

We illustrated our findings by using two real data sets. First, we compared the efficiency of two treatments, *i*.*e*., Natalizumab and Fingolimod, sharing the same indication for active relapsing-remitting multiple sclerosis. Physicians preferentially use Natalizumab in practice for more active disease, indicating possible confounders. Given the absence of a clinical trial with a direct comparison of their efficacy, Barbin *et al*.^53^ recently conducted an observational study. We reused their data. Second, we sought to study barbiturates that can lead to a reduction of the patient functional status. Indeed, barbiturates are suggested in Intensive Care Units (ICU) for the treatment of refractory intracranial pressure increases. However, the use of barbiturates is associated with haemodynamic repercussions that can lead to brain ischaemia and immunodeficiency, which may contribute to the occurrence of infection. These applications were conducted in accordance with the French law relative to clinical noninterventional research. According to the French law on Bioethics (July 29, 1994; August 6, 2004; and July 7, 2011, Public Health Code), the patients’ written informed consent was collected. Moreover, data confidentiality was ensured in accordance with the recommendations of the French commission for data protection (Commission Nationale Informatique et Liberté, CNIL decisions DR-2014-558 and DR-2013-047 for the first and the second application, respectively).

To define the four sets of covariates, we asked experts (D.L. for multiple sclerosis and M.L. for ICU) which covariates were causes of the treatment allocation and which were causes of the outcome, as proposed by VanderWeele and Shpitser^33^. We checked the positivity assumption and the covariate balance (see OSI). We applied B-spline transformations for continuous variables when the log-linearity assumption did not hold.

### Natalizumab versus Fingolimod to prevent relapse in multiple sclerosis patients

The outcome was at least one relapse within one year of treatment initiation. Six hundred and twenty-nine patients from the French national cohort OFSEP were included (www.ofsep.org). The first part of Table 3 presents a description of their baseline characteristics.

**Table 3:** Baseline characteristics of patients of the two studied cohorts.

A - Multiple sclerosis	Overall (n = 629)		First line treatment					Relapse at 1 year				
			Ntz (n = 326)		Fng (n = 303)		*p*	No (n = 478)		Yes (n = 151)		*p*
Patient age, years (mean, sd)	37.0	9.6	36.8	9.9	37.2	9.2	0.6505	37.1	9.7	36.6	9.2	0.5849
Female patient (n, %)	479.0	76.2	254.0	77.9	225.0	74.3	0.2822	367.0	76.8	112.0	74.2	0.5124
Disease duration, years (mean, sd)	8.5	6.4	8.0	6.1	9.0	6.8	0.0505	8.6	6.6	8.2	6.0	0.4809
At least one relapse (n, %)	526.0	83.6	293.0	89.9	233.0	76.9	<0.0001	391.0	81.8	135.0	89.4	0.0277
Gd-enhancing lesion on MRI (n, %)	311.0	49.4	185.0	56.7	126.0	41.6	0.0001	240.0	50.2	71.0	47.0	0.4944
EDSS score >3 (n, %)	288.0	45.8	166.0	50.9	122.0	40.3	0.0074	212.0	44.4	76.0	50.3	0.1986
Previous immunomodulatory treatment (n, %)	556.0	88.4	293.0	89.9	263.0	86.8	0.2284	424.0	88.7	132.0	87.4	0.6672
**B – ICU**	**Overall (n = 252)**		**Barbiturates treatment**					**Favourable GOS at 3 months**				
			**No (n = 178)**		**Yes (n = 74)**		***p***	**No (n = 180)**		**Yes (n = 72)**		***p***
Patient age, years (mean, sd)	47.4	17.4	48.7	17.9	44.1	15.7	0.0565	50.8	16.4	38.7	16.9	<0.0001
Female patient (n, %)	89.0	35.3	58.0	32.6	31.0	41.9	0.1592	68.0	37.8	21.0	29.2	0.1963
Diabetes (n, %)	17.0	6.7	15.0	8.4	2.0	2.7	0.0989	15.0	8.3	2.0	2.8	0.1122
Nosological entity: Severe trauma (n, %)	124.0	49.2	95.0	53.4	29.0	39.2	0.0403	77.0	42.8	47.0	65.3	0.0012
SAP ≤90 mmHg before admission (n, %)	56.0	22.2	36.0	20.2	20.0	27.0	0.2368	46.0	25.6	10.0	13.9	0.0442
Evacuation of subdural or extradural hematoma (n, %)	41.0	16.3	33.0	18.5	8.0	10.8	0.1301	27.0	15.0	14.0	19.4	0.3878
External ventricular drain (n, %)	64.0	25.4	39.0	21.9	25.0	33.8	0.0486	48.0	26.7	16.0	22.2	0.4640
Evacuation of cerebral hematoma or lobectomy (n, %)	42.0	16.7	28.0	15.7	14.0	18.9	0.5362	34.0	18.9	8.0	11.1	0.1345
Decompressive craniectomy (n, %)	27.0	10.7	15.0	8.4	12.0	16.2	0.0686	21.0	11.7	6.0	8.3	0.4396
Blood transfusion before admission (n, %)	34.0	13.5	25.0	14.0	9.0	12.2	0.6903	26.0	14.4	8.0	11.1	0.4841
Pneumonia before increased ICP (n, %)	29.0	11.5	16.0	9.0	13.0	17.6	0.0519	19.0	10.6	10.0	13.9	0.4538
Osmotherapy (n, %)	112.0	44.4	75.0	42.1	37.0	50.0	0.2525	89.0	49.4	23.0	31.9	0.0115
GCS score ≥8	62.0	24.6	39.0	21.9	23.0	31.1	0.1237	37.0	20.6	25.0	34.7	0.0183
Hemoglobin, g/dL (mean, sd)	11.8	2.3	11.7	2.2	12.1	2.5	0.1824	11.8	2.4	11.9	1.9	0.7373
Platelets, counts/mm^3^ (mean, sd)	206.7	78.0	207.4	79.7	205.1	74.2	0.8312	209.0	83.8	200.9	61.1	0.4589
Serum creatinine, mmol/L (mean, sd)	71.1	29.3	71.1	27.6	71.1	33.3	0.9853	72.4	32.6	67.9	18.7	0.2732
Arterial pH (mean, sd)	7.3	0.1	7.3	0.1	7.3	0.1	0.0978	7.3	0.1	7.3	0.1	0.6317
Serum proteins, g/L (mean, sd)	58.2	10.4	57.7	10.6	59.6	9.7	0.1662	58.0	10.7	58.8	9.7	0.5963
Serum urea, mmol/L (mean, sd)	5.0	2.5	5.2	2.7	4.7	1.8	0.1827	5.2	2.3	4.5	2.9	0.0505
PaO_2_/FiO_2_ ratio (mean, sd)	302.7	174.0	292.7	154.7	326.6	212.9	0.1595	282.1	172.4	354.2	168.4	0.0028
SAPS II score (mean, sd)	47.6	11.4	47.6	10.7	47.6	12.9	0.9847	49.9	10.8	41.8	10.7	<0.0001

Ntz: Natalizumab, Fng: Fingolimod, Gd: Gadolinium, MRI: Magnetic Resonance Imaging, EDSS: Expanded Disability Status Scale, SAP: Systolic Arterial Pressure, ICP: Intra-Cranial Pressure, GCS: Glasgow Coma Scale, PaO_2_/FiO_2_: arterial partial Pressure of Oxygen/Fraction of Inspired Oxygen, SAPS II: Simplified Acute Physiology Score II.

All included patients could have received either treatment. Therefore, we sought to estimate the ATE. The first part of Table 4 presents the results according to the different possible methods and covariate sets. The GC, IPTW and TMLE yield similar results regardless of the covariate sets considered. Thus, Fingolimod exhibits lower efficacy than Natalizumab with an OR [95% CI] ranging from 1.50 [1.02; 2.21] for IPTW with the entire set to 1.55 [1.06; 2.28] for GC with the common set. When using FM, the OR ranged from 1.73 [1.19; 2.51] with the outcome set to 1.78 [1.23; 2.56] with the common set. Note that, unlike IPTW, FM does not to balance all covariates in the outcome set with standardised differences higher than 10%.

**Table 4:** Results of the two applications.

application	method	set	${\hat{\pi }}_{0}$	${\hat{\pi }}_{1}$	$\hat{\theta }$	SE	95% CI
**A -** Multiple sclerosis	GC	outcome	20.3	28.2	0.432	0.189	[0.062, 0.802]
		treatment*	20.3	28.3	0.436	0.195	[0.054, 0.819]
		common*	20.3	28.3	0.436	0.195	[0.054, 0.819]
		entire	20.3	28.2	0.431	0.191	[0.056, 0.806]
	IPTW	outcome	21.2	28.8	0.406	0.195	[0.023, 0.789]
		treatment*	20.3	28.2	0.433	0.191	[0.059, 0.808]
		common*	20.3	28.2	0.433	0.191	[0.059, 0.808]
		entire	21.3	28.9	0.406	0.196	[0.022, 0.791]
	TMLE	outcome	21.2	28.8	0.407	0.195	[0.025, 0.790]
		treatment*	20.3	28.2	0.433	0.190	[0.061, 0.806]
		common*	20.3	28.2	0.433	0.190	[0.061, 0.806]
		entire	21.1	28.9	0.410	0.196	[0.026, 0.794]
	FM	outcome	19.1	29.0	0.549	0.189	[0.178, 0.921]
		treatment*	19.9	30.6	0.575	0.187	[0.210, 0.941]
		common*	19.9	30.6	0.575	0.187	[0.210, 0.941]
		entire	21.1	31.9	0.561	0.183	[0.201, 0.920]
**B -** ICU	GC	outcome	66.3	81.1	0.778	0.294	[0.201, 1.354]
		treatment	65.3	81.1	0.824	0.298	[0.240, 1.407]
		common	65.0	81.1	0.836	0.289	[0.270, 1.402]
		entire	66.5	81.1	0.769	0.295	[0.191, 1.347]
	IPTW	outcome	31.0	81.1	0.656	0.356	[−0.042, 1.354]
		treatment	68.2	81.1	0.693	0.355	[−0.002, 1.388]
		common	67.4	81.1	0.729	0.353	[0.038, 1.421]
		entire	69.2	81.1	0.645	0.362	[−0.064, 1.354]
	TMLE	outcome	66.2	79.6	0.692	0.293	[0.118, 1.266]
		treatment	65.4	80.2	0.758	0.288	[0.194, 1.322]
		common	64.8	79.9	0.769	0.298	[0.185, 1.354]
		entire	66.4	79.4	0.668	0.285	[0.109, 1.228]
	FM	outcome	73.8	81.1	0.419	0.342	[−0.252, 1.090]
		treatment	67.2	81.1	0.739	0.337	[0.078, 1.399]
		common	65.1	81.1	0.831	0.336	[0.173, 1.490]
		entire	66.2	81.1	0.782	0.336	[0.123, 1.442]

*Treatment and common sets contain same covariates. *π*_0_: Percentage of event in the Natalizumab (or control) group, *π*_1_: Percentage of event in the Fingolimod (or Barbiturates) group, SE: standard error.

Overall, the confounder-adjusted proportion of patients with at least one relapse within the first year of treatment was lower in the hypothetical world where all patients received Natalizumab (approximately 20% and varying slightly depending on method and set of covariates) than one in which all patients received Fingolimod (approximately 28%). This difference of approximately 8% is clinically meaningful and suggests the superiority of Natalizumab over Fingolimod to prevent relapses at one year. This result was concordant with the recent clinical literature^53,54^.

### Impact of barbiturates in the ICU on the functional status at three months

We define an unfavourable functional outcome by a 3-month Glasgow Outcome Scale (GOS) lower than or equal to 3. We used the data from the French observational cohort AtlanREA (www.atlanrea.org) to estimate the ATT of barbiturates because physicians recommended these drugs to a minority of severe patients. The second part of Table 3 presents the baseline characteristics of the 252 included patients.

The second part of Table 4 presents the results according to the different possible methods and covariate sets. G-computation and TMLE lead to the conclusion of a significant negative effect of barbiturates regardless of the covariate set considered with an OR [95% CI] ranging from 0.43 [0.25; 0.76] for GC with the common set to 0.51 [0.29; 0.90] for TMLE with the entire set. By contrast, the results were discordant when using different covariate sets for IPTW and FM. We report, for instance, OR estimates obtained by FM ranging from 1.520 with the outcome set to 2.300 with the common set. In line with the simulation study, the estimated standard errors were higher for these methods (0.294 and 0.293 for GC and TMLE when the outcome set was considered, respectively) leading to lower power. Note also that standardised differences were higher than 10% for the IPTW with the entire set (see OSI) and for FM with the outcome, the treatment and the entire sets.

Depending on the methods and sets of covariates included, we estimated that from 18% to 20% of patients treated with barbiturates had an unfavourable GOS at three months. If these patients had not received barbiturates, the methods estimate that from 30% to 35% would have had an unfavourable GOS at three months. For the patients, this difference is meaningful but full clinical relevance depends also on the effect of barbiturates on other clinically relevant outcomes, such as death or ventilator-associated pneumonia. However, the results obtained by GC or TMLE differ with those obtained by Majdan *et al*.^55^, who did not find any significant effect of barbiturates on the GOS at six months. Two main methodological reasons can explain this difference: the GOS was at six months rather than three months post-initiation, and the authors used multivariate logistic regression leading to a different estimand.

## Discussion

The aim of this study was to better understand the different sets of covariates to consider when estimating the marginal causal effect.

The results of our simulation study, limited to the studied scenarios, highlight that the use of the outcome set was associated with the lower bias and variance, principally when associated with GC, for both ATE and ATT. As expected, an unmeasured confounder led to increased bias, regardless of method employed. Although we do not report an impact on the variance, the effect’s over- or under-estimation leads to the corresponding over- or under-estimation of power and compromises the validity of the causal inference.

The performance of FM is lower than that of the other studied methods, especially for the variance. Our results were in line with King and Nielsen^56^, who argued for halting the use of PS matching for many reasons such as covariate imbalance, inefficiency, model dependence and bias. Nonetheless, Colson *et al*.^17^ found slightly higher MSE for GC than FM. Their more simplistic scenario, with only two simulated confounders leading to little covariate imbalance, could explain the difference with our results. Moreover, is unclear whether they accounted for the matched nature of the data, as recommended by Austin and Stuart^16^ or Gayat *et al*.^50^.

While DRE offers protection against model misspecification^23,34,36^, our simulation study resulted in the finding that GC was more robust to the choice of the covariate set than the other methods, TMLE included. This result was particularly important when the treatment set was taken into account, which fits with the results of Kang and Schafer^35^: when both the PS and the Q-model were misspecified, DRE had lower performance than GC. Furthermore, GC was associated with lower variance than DRE in several simulation studies^13,17,35^, which accords with our results.

The first application to multiple sclerosis (ATE) illustrated similar results between the studied methods. In contrast, the second application (ATT) to severe trauma or brain-damaged patients showed different results between the methods. In agreement with simulations, the estimations obtained with GC or TMLE were similar in terms of logOR estimation and variance regardless of the covariate set considered. Estimations obtained with IPTW or FM were highly variable, depending on the covariate set employed: some indicated a negative impact of barbiturates and others did not. These results also tended to demonstrate that GC or TMLE had the highest statistical power. Variances obtained by parametric simulations or by bootstrap resampling were similar (results not displayed).

One can, therefore, question the relative predominance of the PS-based approach compared to GC, although there are several potential explanations. First, there appears to be a pre-conceived notion according to which multivariable non-linear regression cannot be used to estimate marginal absolute and relative effects^57^. Indeed, under logistic regression, the mean sample probability of an event is different from the event probability of a subject with the mean sample characteristics. Second, while there is an explicit variance formula for the IPTW^58^, the equivalent is missing for the GC. The variance must be obtained by bootstrapping, simulation or the delta method. Third, several didactic tutorials on PS-based methods can be found, for instance^59–61^.

We still believe that PS-based methods may have value when multivariate modelling is complex, for instance, for multi-state models^62^. In future research, it would be interesting to examine whether the use of potentially better settings would provide equivalent results, such as the Williamson estimator for IPTW^58^, the Abadie-Imbens estimator for PS matching^63^, or bounded the estimation of TMLE, which can also be updated several times^36^. We also emphasise that we did not investigate these methods when the positivity assumption does not hold. Several authors have studied this problem^13,25,35,36,64^. G-computation was less biased than IPTW or DRE except in Porter *et al*.^36^, where the violation of the positivity assumption was also associated with model misspecifications. The robustness of GC to non-positivity could be due to a correct extrapolation into the missing sub-population, which is not feasible with PS^1^. Other perspectives of this work are to extend the problem to i) time-to-event, continuous or multinomial outcomes and ii) multinomial treatment. However, implementing GC using continuous treatment raises many important considerations concerning the research question and resulting inference^64^.

To facilitate its use in practice, we have implemented the estimation of both ATE and ATT, and their 95% CI, from a logistic model in the existing R package entitled *RISCA* (available at cran.r-project.org/web/packages/RISCA). We provide an example of R code in the appendix. Note that the package did not consider the inflation of the type I error rate due to the modelling steps of the Q-model. Users also have to consider novel strategies for post-model selection inference.

In the applications, we classified covariates into sets based on experts knowledge^33^. However, several statistical methods can be useful when no clinical knowledge is available. Heinze *et al*.^65^ proposed a review of the most used, while Witte and Didelez^66^ reviewed strategies specific to causal inference. Alternatively, data-adaptive methods have recently been developed, such as the outcome-adaptive LASSO^67^ to select covariates associated with both the outcome and the treatment allocation. Nevertheless, according to our results, it may be preferable to focus on constructing the best outcome model based on the outcome set. For instance, the consideration of a super learner^68,69^, merging models and modelling machine learning algorithms may represent an exciting perspective^70^.

Finally, we emphasise that the conclusions from our simulation study cannot be generalised to all situations. They are consistent with the current literature on causal inference, but theoretical arguments are missing for generalisation. Notably, our results must be considered in situations where both the PS and the Q-model are correctly specified and where positivity holds.

To conclude, we demonstrate in a simulation study that adjusting for all the covariates causing the outcome improves the estimation of the marginal causal effect (ATE or ATT) of a binary treatment in a binary outcome. Considering only the covariates that are a common cause of both the outcome and the treatment is possible when the number of potential confounders is large. The strategy consisting of considering all available covariates, *i*.*e*., no selection, did not decrease the bias but significantly decreased the power. Amongst the different studied methods, GC had the lowest bias and variance regardless of covariate set considered. Consequently, we recommend that the use of the GC with the outcome set, because of its highest power in all the simulated scenarios. For instance, at least 500 individuals were necessary to achieve a power higher than 80% in ATE, with a theoretical OR at 2, and a percentage of treated subjects at 50%. In ATT, we needed larger sample size to reach a power of 80% because the estimation considers only the treated patients. With 2000 individuals, all the studied methods with the outcome set led to a bias close to zero and a statistical power superior to 95%.

## Supplementary information

Supplementary Information.
