# Supplementary material for: G-computation, propensity score-based methods, and targeted maximum likelihood estimator for causal inference with different covariates sets: a comparative simulation study
Source: Sci Rep. 2020 Jun 8;10:9219. doi: 10.1038/s41598-020-65917-x (PMC7280276; doi:10.1038/s41598-020-65917-x)
Supplement: Supplementary file 1 — Supplementary Information. [file 41598_2020_65917_MOESM1_ESM.pdf]

G-computation, propensity score-based methods, and targeted maximum likelihood estimator for causal inference with different covariates sets: a comparative simulation study

Arthur Chatton[1,2], Florent Le Borgne[1,2], Clémence Leyrat[1,3], Florence Gillaizeau[1,4], Chloé Rousseau[1,4,5], Laetitia Barbin[4], David Laplaud[4,6], Maxime Léger[1,7], Bruno Giraudeau[1,8], and Yohann Foucher[1,4,*]

[1]INSERM UMR 1246 - SPHERE, Université de Nantes, Université de Tours, Nantes, France.
[2]A2COM-IDBC, Pacé, France.
[3]Department of Medical Statistics & Cancer Survival Group, London School of Hygiene and Tropical Medicine, London, UK.
[4]Centre Hospitalier Universitaire de Nantes, Nantes, France.
[5]INSERM CIC1414, CHU Rennes, Rennes, France.
[6]Centre de Recherche en Transplantation et Immunologie INSERM UMR1064, Université de Nantes, Nantes, France.
[7]Département d'Anesthésie-Réanimation, Centre Hospitalier Universitaire d'Angers, Angers, France.
[8]INSERM CIC1415, CHRU de Tours, Tours, France.
[*]Yohann.Foucher@univ-nantes.fr

ABSTRACT

Controlling for confounding bias is crucial in causal inference. Distinct methods are currently employed to mitigate the effects of confounding bias. Each requires the introduction of a set of covariates, which remains difficult to choose, especially regarding the different methods. We conduct a simulation study to compare the relative performance results obtained by using four different sets of covariates (those causing the outcome, those causing the treatment allocation, those causing both the outcome and the treatment allocation, and all the covariates) and four methods: g-computation, inverse probability of treatment weighting, full matching and targeted maximum likelihood estimator. Our simulations are in the context of a binary treatment, a binary outcome and baseline confounders. The simulations suggest that considering all the covariates causing the outcome led to the lowest bias and variance, particularly for g-computation. The consideration of all the covariates did not decrease the bias but significantly reduced the power. We apply these methods to two real-world examples that have clinical relevance, thereby illustrating the real-world importance of using these methods. We propose an R package *RISCA* to encourage the use of g-computation in causal inference.

Supplementary Materials

R code for RISCA use:

```
library(RISCA)

#data simulation
#treatment = 1 if the patients have been the treatment of interest and 0 otherwise
treatment <- rbinom(600, 1, prob=0.5)
covariate <- rnorm(600, 0, 1)
covariate[treatment==1] <- rnorm(sum(treatment==1), 0.3, 1)
outcome <- rbinom(600, 1, prob=1/(1+exp(-2-0.26*treatment-0.7*covariate)))
tab <- data.frame(outcome, treatment, covariate)

#Raw effect of the treatment
```

```
glm.raw <- glm(outcome ~ treatment, data=tab, family = binomial(link=logit))
summary(glm.raw)

#Conditional effect of the treatment
glm.multi <- glm(outcome ~ treatment + covariate, data=tab, family = binomial)
summary(glm.multi)

#Marginal effects of the treatment (ATE)
gc.ate <- GC.Logistic(glm.obj=glm.multi, data=tab, group="treatment", effect="ATE",
var.method="simulations", iterations=1000)

#Sum-up of the 3 ORs
data.frame( raw=exp(glm.raw$coefficients[2]),
conditional=exp(glm.multi$coefficients[2]),
marginal.ate=exp(gc.ate$logOR[,1]) )
```

n	method	set	mean bias				logOR			
			π_0	π_1	$\Delta\pi$	logOR	MSE	VEB	coverage	type I
100	GC	outcome	0.001	0.000	-0.001	-0.002	0.396	-7.1	93.1	5.6
		treatment	0.000	0.001	0.001	0.005	0.457	-7.7	92.8	5.7
		common	0.001	-0.000	-0.001	-0.003	0.419	-5.2	93.6	5.3
		entire	0.000	0.001	0.001	0.005	0.439	-10.9	91.7	6.3
	IPTW	outcome	-0.000	0.001	0.002	0.009	0.455	11.5	97.3	2.7
		treatment	-0.004	0.005	0.008	0.037	0.599	-4.9	94.7	5.3
		common	-0.000	0.001	0.001	0.006	0.462	7.6	96.6	3.5
		entire	-0.004	0.005	0.009	0.040	0.622	-5.6	95.1	5.0
	TMLE	outcome	0.001	0.000	-0.000	0.001	0.432	-14.7	89.8	10.2
		treatment	-0.003	0.005	0.008	0.037	0.549	-23.8	85.0	15.0
		common	0.001	-0.000	-0.001	-0.003	0.452	-9.8	91.4	8.6
		entire	-0.004	0.006	0.009	0.044	0.533	-30.6	81.3	18.7
	FM	outcome	-0.002	0.004	0.006	0.029	0.530	-21.9	88.1	11.9
		treatment	-0.006	0.007	0.013	0.062	0.648	-35.4	79.8	20.1
		common	-0.002	0.003	0.005	0.026	0.534	-22.6	87.7	12.3
		entire	-0.007	0.007	0.014	0.067	0.653	-35.7	79.7	20.2
300	GC	outcome	-0.001	0.000	0.001	0.005	0.217	-2.2	94.7	5.2
		treatment	-0.001	0.001	0.001	0.006	0.251	-2.0	94.5	5.2
		common	-0.001	0.000	0.001	0.004	0.234	-1.7	94.5	5.3
		entire	-0.001	0.001	0.001	0.006	0.235	-3.0	94.4	5.1
	IPTW	outcome	-0.001	0.001	0.002	0.008	0.236	19.3	98.1	1.9
		treatment	-0.002	0.001	0.003	0.012	0.319	5.8	96.2	3.8
		common	-0.001	0.001	0.002	0.008	0.249	12.0	97.0	3.0
		entire	-0.002	0.001	0.003	0.012	0.314	9.2	97.1	2.9
	TMLE	outcome	-0.001	0.000	0.001	0.004	0.230	-3.6	93.9	6.1
		treatment	-0.001	0.001	0.002	0.011	0.301	-10.3	91.3	8.7
		common	-0.001	0.000	0.001	0.004	0.246	-2.5	94.0	6.0
		entire	-0.001	0.001	0.003	0.011	0.281	-12.5	90.3	9.7
	FM	outcome	-0.001	0.001	0.002	0.010	0.285	-17.8	89.4	10.6
		treatment	-0.003	0.002	0.006	0.025	0.374	-37.0	78.5	21.5
		common	-0.001	0.000	0.001	0.007	0.305	-23.1	87.2	12.7
		entire	-0.004	0.003	0.006	0.028	0.359	-34.4	80.5	19.5
500	GC	outcome	-0.000	-0.000	-0.000	-0.000	0.169	-2.4	94.2	5.8
		treatment	-0.000	-0.000	0.000	0.002	0.196	-2.6	94.2	5.7
		common	-0.000	-0.000	0.000	0.000	0.182	-2.1	94.5	5.5
		entire	-0.000	-0.000	0.000	0.001	0.182	-3.0	93.9	5.8
	IPTW	outcome	-0.001	-0.000	0.001	0.003	0.181	18.6	98.0	2.0
		treatment	-0.001	0.000	0.001	0.005	0.244	6.7	96.5	3.5
		common	-0.001	0.000	0.001	0.003	0.192	11.3	97.1	2.9
		entire	-0.001	-0.000	0.001	0.003	0.237	11.3	97.0	3.0
	TMLE	outcome	-0.000	-0.000	-0.000	-0.000	0.178	-3.5	93.6	6.4
		treatment	-0.001	-0.000	0.000	0.002	0.232	-7.2	92.1	8.0
		common	-0.000	-0.000	0.000	0.000	0.191	-2.7	94.3	5.8
		entire	-0.001	-0.000	0.000	0.001	0.216	-8.7	91.5	8.5
	FM	outcome	-0.001	-0.000	0.000	0.002	0.216	-16.3	90.2	9.8
		treatment	-0.003	0.001	0.004	0.015	0.295	-38.5	77.5	22.6
		common	-0.000	-0.000	0.000	0.002	0.260	-30.4	83.2	16.9
		entire	-0.002	0.001	0.003	0.014	0.284	-36.1	79.2	20.8
2000	GC	outcome	-0.000	0.000	0.000	0.001	0.083	-1.0	94.7	5.5
		treatment	0.000	0.000	0.000	0.001	0.096	-0.7	94.8	5.2
		common	0.000	0.000	0.000	0.001	0.090	-0.7	94.9	5.3
		entire	-0.000	0.000	0.000	0.002	0.089	-0.8	94.8	5.4
	IPTW	outcome	-0.000	0.001	0.001	0.004	0.089	20.1	98.1	1.8
		treatment	-0.000	0.000	0.001	0.002	0.116	11.5	97.1	2.9
		common	-0.000	0.001	0.001	0.003	0.094	12.9	97.2	2.8
		entire	-0.000	0.000	0.001	0.003	0.111	16.7	97.7	2.2
	TMLE	outcome	0.000	0.000	0.000	0.001	0.087	-1.3	94.3	5.6
		treatment	0.000	0.000	0.000	0.001	0.111	-0.8	94.7	5.4
		common	0.000	0.000	0.000	0.001	0.093	-0.6	94.7	5.3
		entire	-0.000	0.000	0.001	0.002	0.104	-1.6	94.3	5.7
	FM	outcome	-0.000	0.000	0.000	0.002	0.126	-28.5	84.1	15.9
		treatment	-0.000	0.001	0.001	0.005	0.162	-44.4	72.3	27.7
		common	-0.000	0.000	0.000	0.002	0.197	-54.2	62.7	37.2
		entire	-0.000	0.001	0.001	0.004	0.137	-33.9	80.7	19.4

Simulation results comparing the ATE estimation under the null hypothesis.

n	method	set	mean bias				logOR			
			π_0	π_1	$\Delta\pi$	logOR	MSE	VEB	coverage	power
100	GC	outcome	0.002	-0.000	-0.003	0.000	0.526	-10.9	93.1	6.7
		treatment	0.002	-0.000	-0.003	-0.001	0.584	-10.4	93.1	6.3
		common	0.003	-0.000	-0.003	-0.002	0.555	-9.2	93.6	6.4
		entire	0.001	-0.000	-0.001	0.003	0.562	-13.4	91.8	7.5
	IPTW	outcome	0.003	-0.000	-0.004	-0.010	0.579	6.5	96.6	3.2
		treatment	0.002	-0.000	-0.002	-0.011	0.715	-4.3	94.4	5.5
		common	0.004	-0.000	-0.005	-0.011	0.593	1.6	95.9	4.0
		entire	-0.001	-0.000	0.001	-0.006	0.744	-4.8	94.5	5.5
	TMLE	outcome	0.003	-0.000	-0.004	-0.022	0.696	30.6	95.9	3.9
		treatment	0.003	-0.000	-0.004	-0.037	0.875	210.4	99.2	0.7
		common	0.004	-0.000	-0.004	-0.022	0.709	5.3	94.3	5.6
		entire	0.001	-0.000	-0.001	-0.028	0.884	498.8	99.5	0.5
	FM	outcome	-0.001	-0.000	0.001	0.005	0.662	-20.6	88.9	11.3
		treatment	-0.004	-0.000	0.004	0.010	0.817	-35.3	79.5	20.2
		common	0.000	-0.000	-0.000	0.002	0.658	-20.3	89.0	11.0
		entire	-0.005	-0.000	0.005	0.010	0.835	-36.6	78.5	21.4
300	GC	outcome	-0.000	0.000	0.000	0.004	0.268	-2.2	94.7	5.1
		treatment	-0.000	0.000	0.000	0.003	0.305	-3.0	94.4	5.3
		common	0.000	0.000	0.000	0.003	0.290	-2.5	94.7	5.3
		entire	-0.000	0.000	0.001	0.004	0.282	-3.0	94.7	5.1
	IPTW	outcome	0.000	0.000	-0.000	0.001	0.287	16.0	98.0	2.0
		treatment	-0.002	0.000	0.002	0.008	0.365	4.7	96.1	3.9
		common	0.000	0.000	-0.000	0.001	0.307	7.6	96.8	3.2
		entire	-0.002	0.000	0.003	0.008	0.353	10.4	97.1	2.9
	TMLE	outcome	-0.001	0.000	0.001	0.003	0.347	-11.2	92.1	7.9
		treatment	-0.001	0.000	0.001	-0.001	0.451	71.6	99.3	0.7
		common	-0.001	0.000	0.002	0.005	0.371	-14.7	90.9	9.1
		entire	-0.001	0.000	0.001	-0.003	0.427	106.5	99.6	0.4
	FM	outcome	-0.001	0.000	0.002	0.005	0.338	-13.8	90.9	9.1
		treatment	-0.004	0.000	0.004	0.011	0.444	-34.2	80.3	19.8
		common	-0.002	0.000	0.002	0.007	0.351	-17.0	89.9	10.1
		entire	-0.004	0.000	0.004	0.011	0.437	-33.2	81.8	18.2
500	GC	outcome	0.000	-0.001	-0.001	-0.003	0.203	-0.7	95.0	5.0
		treatment	0.001	-0.001	-0.002	-0.006	0.232	-1.6	94.8	5.1
		common	0.001	-0.001	-0.002	-0.005	0.221	-1.3	94.8	4.9
		entire	0.000	-0.001	-0.001	-0.005	0.215	-1.4	94.8	5.2
	IPTW	outcome	0.001	-0.001	-0.002	-0.008	0.219	16.6	97.9	2.1
		treatment	0.001	-0.001	-0.002	-0.008	0.276	6.2	96.2	3.8
		common	0.001	-0.001	-0.002	-0.009	0.233	8.8	96.7	3.2
		entire	0.000	-0.001	-0.001	-0.007	0.265	11.7	97.0	3.0
	TMLE	outcome	-0.000	-0.001	-0.001	-0.005	0.264	-15.8	90.0	10.0
		treatment	0.001	-0.001	-0.002	-0.012	0.341	51.5	99.0	1.0
		common	-0.000	-0.001	-0.001	-0.004	0.283	-17.2	89.4	10.6
		entire	0.001	-0.001	-0.002	-0.011	0.320	69.4	99.4	0.6
	FM	outcome	-0.000	-0.001	-0.001	-0.004	0.254	-11.7	92.1	7.9
		treatment	-0.001	-0.001	-0.000	-0.004	0.337	-33.2	80.9	19.1
		common	0.000	-0.001	-0.001	-0.005	0.276	-18.5	89.2	10.7
		entire	-0.000	-0.001	-0.001	-0.006	0.324	-30.6	82.7	17.3
2000	GC	outcome	0.000	0.000	0.000	0.000	0.102	-1.7	94.4	5.8
		treatment	0.000	0.000	-0.000	-0.000	0.115	-0.9	94.9	5.2
		common	0.000	0.000	-0.000	-0.000	0.110	-1.1	95.1	4.9
		entire	0.000	0.000	0.000	0.001	0.107	-1.6	94.3	5.8
	IPTW	outcome	0.001	0.000	-0.001	-0.005	0.108	15.9	97.6	2.4
		treatment	0.000	0.000	-0.000	-0.000	0.132	8.8	96.4	3.6
		common	0.001	0.000	-0.001	-0.005	0.115	9.0	96.6	3.4
		entire	0.000	0.000	0.000	0.000	0.127	13.9	97.3	2.7
	TMLE	outcome	0.000	0.000	-0.000	-0.001	0.129	-19.2	88.3	11.7
		treatment	0.000	0.000	0.000	-0.001	0.164	34.4	99.0	1.0
		common	0.000	0.000	-0.000	-0.000	0.138	-18.7	88.6	11.4
		entire	0.000	0.000	0.000	-0.000	0.154	37.2	99.1	0.9
	FM	outcome	0.000	0.000	0.000	0.000	0.129	-13.3	91.0	9.0
		treatment	0.000	0.000	0.000	-0.001	0.196	-43.1	73.4	26.7
		common	0.000	0.000	-0.000	-0.001	0.144	-22.6	87.5	12.6
		entire	0.000	0.000	0.000	-0.001	0.155	-28.1	84.0	16.0

Simulation results comparing the ATT estimation under the null hypothesis.

n	method	set	mean bias				logOR			
			π_0	π_1	$\Delta\pi$	logOR	MSE	VEB	coverage	type I
100	GC	outcome	-0.113	-0.000	0.113	0.471	0.686	-5.1	83.3	16.8
		treatment	-0.123	-0.000	0.123	0.512	0.754	-5.7	83.6	15.9
		common	-0.113	-0.000	0.112	0.468	0.702	-3.6	85.4	14.9
		entire	-0.124	-0.000	0.124	0.516	0.739	-7.9	81.1	18.0
	IPTW	outcome	-0.113	-0.000	0.113	0.471	0.699	9.5	90.7	9.5
		treatment	-0.126	-0.000	0.126	0.526	0.804	1.6	87.6	12.9
		common	-0.113	-0.000	0.112	0.467	0.710	3.9	89.3	10.9
		entire	-0.127	-0.000	0.127	0.531	0.813	4.3	89.0	11.4
	TMLE	outcome	-0.113	-0.000	0.113	0.473	0.771	1.2	87.2	13.1
		treatment	-0.129	-0.000	0.129	0.548	0.929	35.3	93.5	6.8
		common	-0.113	-0.000	0.113	0.471	0.766	4.1	87.9	12.4
		entire	-0.128	-0.000	0.128	0.545	0.933	119.1	96.2	3.9
	FM	outcome	-0.115	-0.000	0.115	0.480	0.764	-12.9	82.1	18.3
		treatment	-0.127	-0.000	0.127	0.536	0.886	-26.3	75.0	25.4
		common	-0.114	-0.000	0.113	0.474	0.765	-13.9	82.3	18.0
		entire	-0.129	-0.000	0.129	0.546	0.891	-26.1	74.0	26.4
300	GC	outcome	-0.115	-0.000	0.115	0.470	0.544	-2.5	57.7	42.2
		treatment	-0.126	-0.000	0.126	0.513	0.598	-3.2	59.3	40.9
		common	-0.115	-0.000	0.115	0.469	0.553	-2.6	62.8	37.8
		entire	-0.126	-0.000	0.125	0.513	0.588	-3.8	54.2	45.2
	IPTW	outcome	-0.115	-0.000	0.115	0.471	0.547	11.3	69.2	31.0
		treatment	-0.129	-0.000	0.129	0.527	0.621	3.0	66.4	33.7
		common	-0.115	-0.000	0.115	0.469	0.555	3.7	68.2	31.9
		entire	-0.129	-0.000	0.129	0.528	0.614	9.3	67.9	32.2
	TMLE	outcome	-0.116	-0.000	0.116	0.475	0.568	1.6	67.9	32.2
		treatment	-0.134	-0.000	0.134	0.551	0.678	-7.8	65.3	34.9
		common	-0.116	-0.000	0.116	0.474	0.576	4.2	72.0	28.1
		entire	-0.134	-0.000	0.134	0.549	0.666	1.8	68.4	31.8
	FM	outcome	-0.115	-0.000	0.115	0.471	0.568	-8.2	62.7	37.5
		treatment	-0.130	-0.000	0.129	0.530	0.652	-23.3	54.3	45.9
		common	-0.115	-0.000	0.115	0.471	0.580	-14.3	61.7	38.4
		entire	-0.131	-0.000	0.130	0.535	0.653	-22.4	53.7	46.4
500	GC	outcome	-0.116	-0.000	0.115	0.470	0.515	-1.9	37.9	61.7
		treatment	-0.127	-0.000	0.126	0.514	0.565	-1.8	39.0	60.4
		common	-0.116	-0.000	0.116	0.471	0.522	-1.7	42.6	57.5
		entire	-0.126	-0.000	0.126	0.513	0.558	-2.3	33.6	65.8
	IPTW	outcome	-0.116	-0.000	0.115	0.470	0.516	11.6	49.8	9.5
		treatment	-0.130	-0.000	0.130	0.530	0.585	4.1	46.8	52.8
		common	-0.116	-0.000	0.116	0.472	0.523	4.4	48.9	50.4
		entire	-0.130	-0.000	0.130	0.528	0.578	11.2	47.7	51.5
	TMLE	outcome	-0.116	-0.000	0.116	0.473	0.529	2.7	50.8	48.5
		treatment	-0.136	-0.000	0.136	0.555	0.631	-12.5	44.0	55.7
		common	-0.117	-0.000	0.117	0.476	0.538	5.2	55.9	43.4
		entire	-0.135	-0.000	0.135	0.551	0.619	-7.5	43.1	56.4
	FM	outcome	-0.116	-0.000	0.116	0.473	0.533	-8.7	44.4	55.1
		treatment	-0.131	-0.000	0.131	0.534	0.608	-23.3	36.8	62.7
		common	-0.117	-0.000	0.117	0.476	0.544	-15.2	44.2	55.4
		entire	-0.131	-0.000	0.130	0.532	0.603	-21.1	37.0	62.5
2000	GC	outcome	-0.115	0.000	0.115	0.465	0.477	-0.6	0.5	99.5
		treatment	-0.126	0.000	0.126	0.509	0.522	-0.7	0.7	99.3
		common	-0.115	0.000	0.115	0.466	0.479	-0.5	1.1	99.0
		entire	-0.126	0.000	0.126	0.508	0.520	-0.8	0.3	99.7
	IPTW	outcome	-0.115	0.000	0.115	0.465	0.477	11.7	1.1	98.9
		treatment	-0.129	0.000	0.129	0.522	0.536	4.5	1.1	98.9
		common	-0.115	0.000	0.115	0.466	0.479	4.8	1.6	98.4
		entire	-0.129	0.000	0.129	0.521	0.534	10.9	0.9	99.1
	TMLE	outcome	-0.116	0.000	0.116	0.468	0.482	4.8	2.4	97.6
		treatment	-0.135	0.000	0.135	0.546	0.565	-16.3	1.7	98.4
		common	-0.116	0.000	0.116	0.469	0.485	5.8	4.1	95.9
		entire	-0.135	0.000	0.135	0.545	0.563	-15.6	0.8	99.2
	FM	outcome	-0.115	0.000	0.115	0.465	0.481	-9.7	2.2	97.8
		treatment	-0.129	0.000	0.129	0.523	0.547	-29.2	2.2	97.8
		common	-0.115	0.000	0.115	0.466	0.484	-15.6	2.9	97.1
		entire	-0.130	0.000	0.130	0.524	0.542	-19.2	1.2	98.8

Simulation results comparing the ATT estimation under the null hypothesis in the presence of an unmeasured confounder.

n	method	set	mean bias				logOR			
			π_0	π_1	$\Delta\pi$	logOR	MSE	VEB	coverage	power
100	GC	outcome	-0.112	-0.002	0.111	0.493	0.716	-2.7	86.3	50.7
		treatment	-0.123	-0.002	0.122	0.538	0.786	-3.6	85.5	46.0
		common	-0.112	-0.002	0.111	0.492	0.735	-1.7	87.8	45.6
		entire	-0.123	-0.002	0.121	0.538	0.768	-5.5	83.5	50.7
	IPTW	outcome	-0.113	-0.002	0.111	0.494	0.727	11.7	92.7	36.6
		treatment	-0.126	-0.002	0.125	0.553	0.837	2.9	89.1	36.3
		common	-0.113	-0.002	0.111	0.494	0.743	5.6	90.8	38.4
		entire	-0.126	-0.002	0.124	0.553	0.838	6.0	90.4	33.9
	TMLE	outcome	-0.113	-0.002	0.111	0.498	0.794	16.1	94.3	26.3
		treatment	-0.129	-0.002	0.128	0.575	0.955	38.1	97.1	16.6
		common	-0.114	-0.002	0.112	0.499	0.794	19.6	93.9	24.7
		entire	-0.128	-0.002	0.126	0.570	0.953	110.0	98.4	9.7
	FM	outcome	-0.114	-0.002	0.113	0.503	0.787	-9.0	85.0	45.1
		treatment	-0.128	-0.002	0.127	0.567	0.911	-22.5	76.6	49.4
		common	-0.115	-0.002	0.113	0.504	0.795	-10.6	84.3	44.2
		entire	-0.128	-0.002	0.126	0.566	0.921	-23.8	76.2	49.6
300	GC	outcome	-0.118	0.001	0.119	0.476	0.557	-1.0	62.7	94.6
		treatment	-0.128	0.001	0.129	0.517	0.607	-1.2	63.1	91.0
		common	-0.117	0.001	0.118	0.474	0.563	0.1	67.1	91.5
		entire	-0.128	0.001	0.129	0.520	0.601	-2.6	58.9	94.5
	IPTW	outcome	-0.117	0.001	0.119	0.476	0.558	12.1	73.5	88.9
		treatment	-0.131	0.001	0.132	0.530	0.629	4.3	69.6	85.3
		common	-0.117	0.001	0.118	0.474	0.564	6.1	71.6	88.2
		entire	-0.131	0.001	0.132	0.532	0.624	10.0	71.2	85.5
	TMLE	outcome	-0.118	0.001	0.119	0.479	0.579	16.8	80.9	78.8
		treatment	-0.135	0.001	0.137	0.551	0.685	1.0	75.7	76.8
		common	-0.118	0.001	0.119	0.478	0.584	20.6	84.1	73.0
		entire	-0.136	0.001	0.137	0.552	0.676	9.7	78.6	74.6
	FM	outcome	-0.118	0.001	0.119	0.478	0.580	-6.1	66.5	88.5
		treatment	-0.131	0.001	0.132	0.532	0.658	-20.2	58.4	87.3
		common	-0.117	0.001	0.118	0.475	0.589	-11.3	65.2	86.8
		entire	-0.132	0.001	0.133	0.538	0.664	-20.6	57.7	87.7
500	GC	outcome	-0.115	-0.001	0.114	0.457	0.509	-1.5	45.4	99.4
		treatment	-0.125	-0.001	0.125	0.500	0.556	-0.5	46.3	99.0
		common	-0.115	-0.001	0.114	0.456	0.513	-0.1	50.8	98.9
		entire	-0.126	-0.001	0.125	0.501	0.552	-2.1	40.7	99.4
	IPTW	outcome	-0.115	-0.001	0.114	0.457	0.509	11.1	56.6	98.7
		treatment	-0.129	-0.001	0.128	0.513	0.574	5.0	53.3	97.7
		common	-0.115	-0.001	0.114	0.456	0.514	5.4	56.0	98.3
		entire	-0.129	-0.001	0.128	0.514	0.571	9.8	53.8	98.0
	TMLE	outcome	-0.115	-0.001	0.114	0.458	0.521	18.1	69.4	95.6
		treatment	-0.134	-0.001	0.133	0.536	0.618	-2.3	57.4	94.8
		common	-0.115	-0.001	0.114	0.459	0.527	20.5	73.6	93.2
		entire	-0.134	-0.001	0.133	0.535	0.611	1.4	57.2	95.5
	FM	outcome	-0.115	-0.001	0.114	0.458	0.525	-7.5	51.9	98.0
		treatment	-0.129	-0.001	0.129	0.517	0.597	-20.3	43.0	97.6
		common	-0.114	-0.001	0.113	0.455	0.530	-12.7	51.9	96.9
		entire	-0.129	-0.001	0.129	0.517	0.596	-19.7	43.1	97.6
2000	GC	outcome	-0.115	0.000	0.116	0.466	0.479	-1.0	0.7	100.0
		treatment	-0.126	0.000	0.126	0.510	0.524	-0.7	0.9	100.0
		common	-0.115	0.000	0.115	0.466	0.480	-0.5	1.6	100.0
		entire	-0.126	0.000	0.126	0.510	0.523	-0.7	0.3	100.0
	IPTW	outcome	-0.115	0.000	0.115	0.465	0.478	10.7	1.7	100.0
		treatment	-0.129	0.000	0.130	0.523	0.539	4.2	1.6	100.0
		common	-0.115	0.000	0.115	0.466	0.480	4.3	2.3	100.0
		entire	-0.129	0.000	0.130	0.523	0.537	10.6	1.2	100.0
	TMLE	outcome	-0.116	0.000	0.116	0.467	0.483	18.0	5.4	100.0
		treatment	-0.135	0.000	0.136	0.548	0.568	-6.6	2.6	100.0
		common	-0.116	0.000	0.116	0.469	0.486	18.6	8.3	100.0
		entire	-0.135	0.000	0.135	0.546	0.565	-4.6	1.7	100.0
	FM	outcome	-0.115	0.000	0.116	0.466	0.484	-8.4	2.9	100.0
		treatment	-0.130	0.000	0.130	0.524	0.548	-26.4	3.0	100.0
		common	-0.115	0.000	0.115	0.466	0.485	-13.9	3.7	100.0
		entire	-0.130	0.000	0.130	0.525	0.543	-16.3	1.4	100.0

Simulation results comparing the ATT estimation under the alternative hypothesis in the presence of an unmeasured confounder.

n	method	set	mean bias				logOR			
			π_0	π_1	$\Delta\pi$	logOR	MSE	VEB	coverage	type I
100	GC	outcome	-0.058	0.057	0.115	0.479	0.628	-6.4	74.9	23.4
		treatment	-0.065	0.065	0.131	0.546	0.716	-7.7	74.3	23.3
		common	-0.058	0.057	0.115	0.479	0.643	-4.9	78.0	21.3
		entire	-0.065	0.066	0.131	0.547	0.704	-10.0	70.8	26.0
	IPTW	outcome	-0.058	0.057	0.115	0.481	0.638	9.6	85.0	15.0
		treatment	-0.065	0.065	0.130	0.548	0.754	-1.6	80.4	19.6
		common	-0.058	0.057	0.115	0.480	0.650	3.1	83.1	16.9
		entire	-0.065	0.065	0.130	0.550	0.753	1.7	81.8	18.1
	TMLE	outcome	-0.058	0.057	0.115	0.479	0.635	-6.5	75.5	24.4
		treatment	-0.065	0.064	0.130	0.548	0.746	-11.8	74.2	25.8
		common	-0.058	0.057	0.115	0.480	0.649	-4.1	79.4	20.6
		entire	-0.065	0.065	0.130	0.549	0.735	-16.9	68.7	31.3
	FM	outcome	-0.058	0.056	0.115	0.483	0.687	-15.1	75.3	24.7
		treatment	-0.068	0.067	0.135	0.575	0.823	-28.8	65.1	34.9
		common	-0.060	0.057	0.116	0.491	0.707	-18.3	74.1	25.9
		entire	-0.068	0.067	0.135	0.574	0.818	-28.0	65.3	34.7
300	GC	outcome	-0.056	0.056	0.113	0.462	0.513	-1.4	45.0	55.1
		treatment	-0.064	0.064	0.128	0.528	0.586	-1.5	44.8	55.1
		common	-0.056	0.056	0.112	0.461	0.518	-0.6	50.8	49.7
		entire	-0.064	0.064	0.128	0.528	0.580	-2.6	38.4	61.1
	IPTW	outcome	-0.057	0.056	0.113	0.462	0.515	12.7	57.0	43.0
		treatment	-0.064	0.063	0.127	0.524	0.592	4.0	54.9	45.1
		common	-0.056	0.056	0.112	0.461	0.520	5.9	56.5	43.6
		entire	-0.064	0.063	0.127	0.525	0.587	9.6	56.0	44.1
	TMLE	outcome	-0.057	0.056	0.112	0.462	0.514	-1.2	45.9	54.2
		treatment	-0.064	0.063	0.127	0.522	0.589	-3.0	49.8	50.3
		common	-0.056	0.056	0.112	0.461	0.519	-0.2	51.8	48.3
		entire	-0.064	0.063	0.127	0.522	0.583	-4.6	44.1	56.0
	FM	outcome	-0.057	0.056	0.113	0.465	0.534	-10.3	50.2	49.9
		treatment	-0.064	0.064	0.129	0.532	0.624	-27.4	41.2	58.9
		common	-0.056	0.056	0.112	0.464	0.562	-25.4	49.8	50.3
		entire	-0.065	0.064	0.129	0.534	0.622	-25.6	41.2	58.9
500	GC	outcome	-0.057	0.056	0.113	0.462	0.494	-2.1	23.2	76.8
		treatment	-0.065	0.064	0.129	0.528	0.564	-2.0	22.6	77.3
		common	-0.057	0.056	0.113	0.462	0.498	-1.9	29.1	71.2
		entire	-0.065	0.064	0.129	0.527	0.559	-2.5	17.5	82.1
	IPTW	outcome	-0.057	0.056	0.113	0.462	0.494	11.3	33.3	66.6
		treatment	-0.065	0.063	0.128	0.524	0.565	3.2	32.4	67.4
		common	-0.057	0.056	0.113	0.462	0.498	4.3	33.8	65.9
		entire	-0.065	0.063	0.128	0.524	0.562	8.9	32.3	67.5
	TMLE	outcome	-0.057	0.056	0.113	0.462	0.494	-1.6	24.0	75.8
		treatment	-0.065	0.063	0.127	0.523	0.563	-2.6	27.9	71.9
		common	-0.057	0.056	0.113	0.462	0.498	-1.4	29.6	70.1
		entire	-0.064	0.063	0.127	0.522	0.559	-3.2	22.4	77.4
	FM	outcome	-0.057	0.056	0.113	0.461	0.507	-13.1	31.2	68.6
		treatment	-0.065	0.064	0.128	0.529	0.589	-29.7	25.2	74.7
		common	-0.057	0.056	0.113	0.463	0.540	-34.1	35.4	64.5
		entire	-0.065	0.063	0.128	0.528	0.582	-25.3	23.8	76.1
2000	GC	outcome	-0.057	0.056	0.113	0.460	0.468	-0.9	0.0	100.0
		treatment	-0.065	0.064	0.128	0.525	0.534	-0.7	0.0	100.0
		common	-0.057	0.056	0.113	0.460	0.469	-0.7	0.1	99.9
		entire	-0.064	0.064	0.128	0.525	0.533	-0.9	0.0	100.0
	IPTW	outcome	-0.057	0.056	0.113	0.459	0.468	12.1	0.1	99.9
		treatment	-0.064	0.063	0.127	0.520	0.530	4.6	0.2	99.8
		common	-0.057	0.056	0.113	0.459	0.468	5.0	0.2	99.8
		entire	-0.064	0.063	0.127	0.520	0.529	11.0	0.0	100.0
	TMLE	outcome	-0.057	0.056	0.113	0.459	0.467	-0.8	0.0	100.0
		treatment	-0.064	0.063	0.127	0.518	0.528	-1.0	0.1	99.9
		common	-0.057	0.056	0.113	0.459	0.468	-0.7	0.1	99.9
		entire	-0.064	0.063	0.127	0.518	0.527	-0.9	0.0	100.0
	FM	outcome	-0.057	0.056	0.112	0.459	0.479	-34.3	1.9	98.1
		treatment	-0.064	0.063	0.127	0.521	0.549	-48.2	2.5	97.5
		common	-0.056	0.056	0.113	0.462	0.515	-60.0	10.6	89.4
		entire	-0.064	0.063	0.127	0.518	0.532	-24.9	0.3	99.7

Simulation results comparing the ATE estimation under the null hypothesis in the presence of an unmeasured confounder.

n	method	set	mean bias				logOR			
			π_0	π_1	$\Delta\pi$	logOR	MSE	VEB	coverage	power
100	GC	outcome	-0.056	0.057	0.113	0.478	0.634	-6.9	76.2	67.5
		treatment	-0.065	0.065	0.129	0.551	0.727	-7.9	75.5	62.2
		common	-0.057	0.057	0.113	0.479	0.650	-5.2	79.2	62.8
		entire	-0.065	0.065	0.129	0.550	0.714	-10.6	71.6	67.0
	IPTW	outcome	-0.057	0.056	0.113	0.480	0.646	8.1	85.4	54.3
		treatment	-0.064	0.065	0.128	0.556	0.769	-2.4	80.9	51.8
		common	-0.057	0.056	0.113	0.482	0.657	2.9	84.2	55.5
		entire	-0.063	0.065	0.128	0.556	0.773	-0.7	82.0	50.1
	TMLE	outcome	-0.056	0.056	0.113	0.478	0.642	-7.0	77.3	66.5
		treatment	-0.063	0.064	0.128	0.552	0.757	-12.2	75.1	60.0
		common	-0.057	0.056	0.113	0.481	0.657	-4.1	80.6	60.8
		entire	-0.063	0.065	0.128	0.553	0.748	-17.6	69.6	66.6
	FM	outcome	-0.058	0.057	0.115	0.497	0.710	-16.7	75.2	62.1
		treatment	-0.065	0.066	0.130	0.575	0.832	-29.0	66.6	64.9
		common	-0.056	0.057	0.114	0.491	0.713	-18.5	75.0	61.4
		entire	-0.065	0.066	0.131	0.577	0.830	-28.4	66.2	65.3
300	GC	outcome	-0.057	0.058	0.115	0.480	0.532	-1.9	43.5	98.7
		treatment	-0.065	0.066	0.131	0.548	0.608	-2.8	42.9	97.7
		common	-0.057	0.058	0.115	0.480	0.539	-1.9	48.7	97.8
		entire	-0.065	0.066	0.131	0.547	0.600	-2.9	37.1	98.9
	IPTW	outcome	-0.057	0.058	0.115	0.480	0.533	12.4	55.3	97.6
		treatment	-0.064	0.066	0.130	0.546	0.615	3.0	53.1	94.2
		common	-0.057	0.058	0.115	0.481	0.541	4.7	54.4	96.6
		entire	-0.064	0.066	0.130	0.545	0.607	9.6	54.8	94.8
	TMLE	outcome	-0.057	0.058	0.115	0.480	0.532	-1.0	44.9	98.7
		treatment	-0.064	0.066	0.130	0.545	0.612	-3.3	48.0	96.2
		common	-0.057	0.058	0.115	0.481	0.540	-1.1	50.1	97.6
		entire	-0.064	0.066	0.130	0.543	0.603	-3.4	42.8	97.9
	FM	outcome	-0.057	0.058	0.115	0.481	0.552	-11.9	48.1	96.6
		treatment	-0.065	0.066	0.132	0.556	0.652	-29.0	40.2	95.3
		common	-0.057	0.058	0.115	0.486	0.588	-27.4	47.6	93.1
		entire	-0.065	0.067	0.132	0.557	0.644	-25.5	39.1	96.0
300	GC	outcome	-0.057	0.056	0.113	0.469	0.501	-1.0	23.0	100.0
		treatment	-0.065	0.064	0.129	0.536	0.573	-2.0	22.8	99.9
		common	-0.057	0.056	0.113	0.468	0.505	-1.0	29.0	99.9
		entire	-0.065	0.064	0.129	0.536	0.569	-1.9	17.5	100.0
	IPTW	outcome	-0.057	0.056	0.113	0.469	0.501	12.6	32.9	100.0
		treatment	-0.064	0.064	0.127	0.531	0.573	3.6	32.9	99.6
		common	-0.057	0.056	0.113	0.468	0.505	5.1	33.7	99.9
		entire	-0.064	0.064	0.127	0.532	0.569	10.2	32.6	99.6
	TMLE	outcome	-0.057	0.056	0.113	0.468	0.501	-0.4	23.7	100.0
		treatment	-0.064	0.063	0.127	0.530	0.571	-2.1	28.0	99.8
		common	-0.057	0.056	0.113	0.468	0.505	-0.5	29.3	99.9
		entire	-0.064	0.064	0.127	0.530	0.566	-1.9	22.9	99.9
	FM	outcome	-0.057	0.056	0.113	0.471	0.518	-14.2	30.3	99.8
		treatment	-0.064	0.064	0.128	0.537	0.598	-29.3	25.0	99.5
		common	-0.057	0.056	0.113	0.475	0.555	-35.3	34.9	98.4
		entire	-0.064	0.064	0.128	0.537	0.592	-25.2	23.4	99.6
300	GC	outcome	-0.058	0.057	0.115	0.474	0.482	-1.3	0.0	100.0
		treatment	-0.066	0.065	0.130	0.541	0.550	-1.3	0.0	100.0
		common	-0.058	0.057	0.115	0.474	0.483	-0.7	0.1	100.0
		entire	-0.066	0.065	0.131	0.541	0.550	-1.7	0.0	100.0
	IPTW	outcome	-0.058	0.057	0.115	0.474	0.482	11.3	0.1	100.0
		treatment	-0.065	0.064	0.129	0.536	0.547	3.7	0.1	100.0
		common	-0.058	0.057	0.114	0.473	0.483	4.9	0.1	100.0
		entire	-0.065	0.064	0.129	0.537	0.546	9.3	0.1	100.0
	TMLE	outcome	-0.058	0.057	0.115	0.474	0.482	-1.1	0.0	100.0
		treatment	-0.065	0.064	0.129	0.534	0.545	-1.6	0.1	100.0
		common	-0.058	0.057	0.114	0.473	0.483	-0.7	0.1	100.0
		entire	-0.065	0.064	0.129	0.535	0.544	-2.1	0.0	100.0
	FM	outcome	-0.058	0.057	0.115	0.475	0.497	-36.3	2.0	100.0
		treatment	-0.065	0.064	0.129	0.539	0.569	-49.2	2.2	100.0
		common	-0.058	0.057	0.115	0.479	0.534	-60.7	9.7	100.0
		entire	-0.065	0.064	0.129	0.535	0.549	-26.0	0.2	100.0

Simulation results comparing the ATE estimation under the alternative hypothesis in the presence of an unmeasured confounder.

	Relapse at 1 year	Fist-line treatment allocation*	Both	Nothing
Female patient				X
At least one relapse 1 year before treatment initiation			X	
Gd-enhancing lesion on MRI	X			
EDSS score >3			X	
Previous immunomodulatory treatment				X
Patient age	X			
Disease duration	X			

Classification of covariates in the multiple sclerosis application according to an expert knowledge. *: No covariate is only associated with the treatment allocation, treatment and common sets are the same.

	Favourable GOS at 3 months	Barbiturates allocation	Both	Nothing
Female patient				X
Diabetes			X	
Nosological entity: Severe trauma			X	
SAP $\leq$ 90 mmHg before admission*	X			
Evacuation of subdural or extradural hematoma		X		
External ventricular drain		X		
Evacuation of cerebral hematoma or lobectomy			X	
Decompressive craniectomy			X	
Blood transfusion before admission	X			
Pneumonia before increased ICP			X	
Osmotherapy	X			
GCS score $\geq$ 8*			X	
Patient age*			X	
Haemoglobin	X			
Platelets				X
Serum creatinine				X
Arterial pH			X	
Serum proteins				X
Serum urea*				X
PaO2/FiO2 ratio			X	
SAPS II score			X	

Classification of covariates in the intensive care unit application according to an expert knowledge. *: No include into a covariate set due to the association with the SAPS II Score.

Set	Characteristics	Overall		Fingolimod		Natalizumab		STD (%)
Outcome	At least one relapse (n, %)	526.9	83.8	273.4	83.9	253.5	83.7	0.5
	EDSS score > 3 (n, %)	286.2	45.5	148.6	45.6	137.6	45.5	0.3
	Gd-enhancing lesion on MRI (n, %)	310.0	49.3	161.1	49.4	148.9	49.2	0.5
	Patient age, years (mean, sd)	37.1	9.6	37.2	9.9	37.1	9.3	1.2
	Disease duration, years (mean, sd)	8.5	6.4	8.6	6.2	8.5	6.5	0.2
Treatment*	At least one relapse (n, %)	526.2	83.7	272.7	83.7	253.5	83.6	0.1
	EDSS score > 3 (n, %)	288.5	45.9	149.5	45.9	139.0	45.8	0.1
Common*	At least one relapse (n, %)	526.2	83.7	272.7	83.7	253.5	83.6	0.1
	EDSS score > 3 (n, %)	288.5	45.9	149.5	45.9	139.0	45.8	0.1
Entire	Female patient (n, %)	481.4	76.6	250.2	76.8	231.2	76.4	0.9
	At least one relapse (n, %)	527.8	84.0	274.3	84.2	253.5	83.8	1.1
	Gd-enhancing lesion on MRI (n, %)	308.8	49.1	160.4	49.2	148.4	49.0	0.4
	EDSS score > 3 (n, %)	285.2	45.4	148.1	45.5	137.1	45.3	0.3
	Previous IMT (n, %)	555.7	88.4	288.2	88.5	267.5	88.4	0.2
	Patient age, years (mean, sd)	37.1	9.7	37.2	10.0	37.1	9.3	1.3
	Disease duration, years (mean, sd)	8.6	6.4	8.6	6.4	8.6	6.4	0.6

The PS-adjusted samples for weighted analysis of the relapsing-remitting multiple sclerosis relapse in the year after the treatment initiation according to the covariate sets. Qualitative characteristics are presented by using the weighted effective (n) and the weighted percentage. Continuous characteristics are presented with weighted mean following by weighted standard deviation (sd). STD: Standardised differences in %, EDSS: Expanded Disability Status Scale, Gd: Gadolinium, MRI: Magnetic Resonance Imaging, and IMT: immunomodulatory treatment. *: No covariate is only associated with the treatment allocation, treatment and common sets are the same.

Set	Characteristics	Overall		Fingolimod		Natalizumab		STD (%)
Outcome	At least one relapse (n, %)	481.1	76.5	248.1	76.1	233.0	76.9	1.8
	EDSS score > 3 (n, %)	236.2	37.6	114.2	35.0	122.0	40.3	10.8
	Gd-enhancing lesion on MRI (n, %)	271.6	43.2	145.6	44.7	126.0	41.6	6.2
	Patient age, years (mean, sd)	36.6	9.6	36.0	9.8	37.2	9.2	12.2
	Disease duration, years (mean, sd)	8.6	6.3	8.1	5.9	9.0	6.8	14.4
Treatment*	At least one relapse (n, %)	483.7	76.9	250.7	76.9	233.0	76.9	0.0
	EDSS score > 3 (n, %)	253.3	40.3	131.3	40.3	122.0	40.3	0.0
Common*	At least one relapse (n, %)	483.7	76.9	250.7	76.9	233.0	76.9	0.0
	EDSS score > 3 (n, %)	253.3	40.3	131.3	40.3	122.0	40.3	0.0
Entire	Female patient (n, %)	483.8	76.9	258.8	79.4	225.0	74.3	12.2
	At least one relapse (n, %)	475.9	75.7	242.9	74.5	233.0	76.9	5.5
	Gd-enhancing lesion on MRI (n, %)	266.5	42.4	140.5	43.1	126.0	41.6	3.1
	EDSS score > 3 (n, %)	255.8	40.7	133.8	41.1	122.0	40.3	1.6
	Previous IMT (n, %)	545.6	86.7	282.6	86.7	263.0	86.8	0.3
	Patient age, years (mean, sd)	37.1	9.3	37.0	9.3	37.2	9.2	1.6
	Disease duration, years (mean, sd)	8.9	6.5	8.8	6.3	9.0	6.8	3.5

The PS-adjusted samples for matched (FM) analysis of the relapsing-remitting multiple sclerosis relapse in the year after the treatment initiation according to the covariate sets. Qualitative characteristics are presented by using the matched effective (n) and the matched percentage. Continuous characteristics are presented with matched mean following by matched standard deviation (sd). STD: Standardised differences in %, EDSS: Expanded Disability Status Scale, Gd: Gadolinium, MRI: Magnetic Resonance Imaging, and IMT: immunomodulatory treatment. *: No covariate is only associated with the treatment allocation, treatment and common sets are the same.

Set	Characteristics	Overall		Barbiturates		Control		STD (%)
Outcome	Diabetes (n, %)	4.1	2.7	2.1	2.7	2.0	2.7	0.2
	Evacuation of cerebral hematoma or lobectomy (n, %)	29.2	19.6	15.2	20.3	14.0	18.9	3.4
	Decompressive craniectomy (n, %)	26.1	17.5	14.1	18.8	12.0	16.2	6.9
	Blood transfusion before admission (n, %)	17.0	11.4	8.0	10.7	9.0	12.2	4.6
	Osmotherapy (n, %)	74.2	49.9	37.2	49.8	37.0	50.0	0.4
	Pneumonia before increased ICP (n, %)	26.3	17.7	13.3	17.8	13.0	17.6	0.7
	Nosological entity: Severe trauma (n, %)	56.5	38.0	27.5	36.8	29.0	39.2	4.9
	Haemoglobin, g/dL (mean, sd)	12.1	2.3	12.2	2.2	12.1	2.5	0.6
	Arterial pH (mean, sd)	7.3	0.1	7.3	0.1	7.3	0.1	2.2
	PaO2/FiO2 ratio (mean, sd)	322.3	193.0	317.9	172.2	326.6	212.9	4.5
	SAPS II score (mean, sd)	47.9	11.5	48.3	10.1	47.6	12.9	6.3
Treatment	Diabetes (n, %)	4.0	2.7	2.0	2.7	2.0	2.7	0.3
	Evacuation of subdural or extradural hematoma (n, %)	16.2	10.8	8.2	10.9	8.0	10.8	0.2
	External ventricular drain (n, %)	52.1	34.9	27.1	35.9	25.0	33.8	4.5
	Evacuation of cerebral hematoma or lobectomy (n, %)	28.8	19.3	14.8	19.6	14.0	18.9	1.8
	Decompressive craniectomy (n, %)	25.9	17.4	13.9	18.5	12.0	16.2	6.1
	Pneumonia before increased ICP (n, %)	27.0	18.1	14.0	18.5	13.0	17.6	2.5
	Nosological entity: Severe trauma (n, %)	57.1	38.2	28.1	37.3	29.0	39.2	3.9
	Arterial pH (mean, sd)	7.3	0.1	7.3	0.1	7.3	0.1	1.9
	PaO2/FiO2 ratio (mean, sd)	323.3	195.9	320.1	178.8	326.6	212.9	3.3
	SAPS II score (mean, sd)	47.8	11.5	48.0	10.0	47.6	12.9	4.2
Common	Diabetes (n, %)	4.0	2.7	2.0	2.7	2.0	2.7	0.1
	Evacuation of cerebral hematoma or lobectomy (n, %)	28.9	19.4	14.9	19.8	14.0	18.9	2.3
	Decompressive craniectomy (n, %)	25.3	17.0	13.3	17.8	12.0	16.2	4.1
	Pneumonia before increased ICP (n, %)	26.5	17.8	13.5	18.0	13.0	17.6	1.2
	Nosological entity: Severe trauma (n, %)	57.2	38.4	28.2	37.6	29.0	39.2	3.3
	Arterial pH (mean, sd)	7.3	0.1	7.3	0.1	7.3	0.1	3.6
	PaO2/FiO2 ratio (mean, sd)	323.8	192.8	321.1	172.0	326.6	212.9	2.9
	SAPS II score (mean, sd)	47.9	11.6	48.3	10.1	47.6	12.9	6.7
Entire	Female patients (n, %)	66.7	44.8	35.7	47.7	31.0	41.9	11.8
	Diabetes (n, %)	4.0	2.7	2.0	2.7	2.0	2.7	0.2
	Evacuation of subdural or extradural hematoma (n, %)	15.4	10.3	7.4	9.9	8.0	10.8	3.0
	External ventricular drain (n, %)	52.3	35.2	27.3	36.5	25.0	33.8	5.8
	Evacuation of cerebral hematoma or lobectomy (n, %)	30.6	20.5	16.6	22.2	14.0	18.9	8.0
	Decompressive craniectomy (n, %)	27.1	18.2	15.1	20.2	12.0	16.2	10.3
	Blood transfusion before admission (n, %)	16.6	11.1	7.6	10.1	9.0	12.2	6.4
	Osmotherapy (n, %)	75.3	50.6	38.3	51.2	37.0	50.0	2.4
	Pneumonia before increased ICP (n, %)	26.2	17.6	13.2	17.6	13.0	17.6	0.1
	Nosological entity: Severe trauma (n, %)	56.0	37.6	27.0	36.1	29.0	39.2	6.4
	Haemoglobin, g/dL (mean, sd)	12.2	2.3	12.2	2.2	12.1	2.5	4.5
	Platelets, counts/mm^3 (mean, sd)	208.8	82.7	212.5	90.7	205.1	74.2	9.0
	Serum creatinine, mmol/L (mean, sd)	70.6	30.9	70.2	28.6	71.1	33.3	2.7
	Arterial pH (mean, sd)	7.3	0.1	7.3	0.1	7.3	0.1	0.5
	Serum proteins, g/L (mean, sd)	59.4	9.5	59.2	9.4	59.6	9.7	4.2
	PaO2/FiO2 ratio (mean, sd)	317.9	196.8	309.3	180.5	326.6	212.9	8.8
	SAPS II score (mean, sd)	47.9	11.5	48.2	10.1	47.6	12.9	5.5

The PS-adjusted samples for weighted analysis of dichotomised Glasgow Outcome Scale score at 3 months according to the covariate sets. Qualitative characteristics are presented by using the weighted effective (n) and the weighted percentage. Continuous characteristics are presented with weighted mean following by weighted standard deviation (sd). STD: Standardised differences in %, SAPS: Simplified Acute Physiology Score, ICP: Intracranial Pressure, PaO2: arterial partial Pressure of Oxygen, and FiO2: Fraction of Inspired Oxygen.

Set	Characteristics	Overall		Barbiturates		Control		STD (%)
Outcome	Diabetes (n, %)	5.7	2.3	3.7	2.1	2.0	2.7	4.0
	Evacuation of cerebral hematoma or lobectomy (n, %)	42.2	16.7	28.2	15.8	14.0	18.9	8.1
	Decompressive craniectomy (n, %)	39.5	15.7	27.5	15.5	12.0	16.2	2.0
	Blood transfusion before admission (n, %)	28.2	11.2	19.2	10.8	9.0	12.2	4.3
	Osmotherapy (n, %)	121.8	48.3	84.8	47.7	37.0	50.0	4.7
	Pneumonia before increased ICP (n, %)	45.9	18.2	32.9	18.5	13.0	17.6	2.3
	Nosological entity: Severe trauma (n, %)	95.0	37.7	66.0	37.1	29.0	39.2	4.4
	Haemoglobin, g/dL (mean, sd)	12.1	2.3	12.1	2.3	12.1	2.5	2.1
	Arterial pH (mean, sd)	7.4	0.1	7.4	0.1	7.3	0.1	7.6
	PaO2/FiO2 ratio (mean, sd)	313.1	174.0	307.4	155.4	326.6	212.9	10.3
	SAPS II score (mean, sd)	48.4	11.1	48.7	10.3	47.6	12.9	9.8
Treatment	Diabetes (n, %)	5.1	2.0	3.1	1.7	2.0	2.7	6.6
	Evacuation of subdural or extradural hematoma (n, %)	28.2	11.2	20.2	11.4	8.0	10.8	1.7
	External ventricular drain (n, %)	74.0	29.4	49.0	27.5	25.0	33.8	13.6
	Evacuation of cerebral hematoma or lobectomy (n, %)	43.5	17.3	29.5	16.6	14.0	18.9	6.1
	Decompressive craniectomy (n, %)	45.6	18.1	33.6	18.9	12.0	16.2	6.9
	Pneumonia before increased ICP (n, %)	41.5	16.5	28.5	16.0	13.0	17.6	4.2
	Nosological entity: Severe trauma (n, %)	97.1	38.5	68.1	38.3	29.0	39.2	1.9
	Arterial pH (mean, sd)	7.4	0.1	7.4	0.1	7.3	0.1	12.4
	PaO2/FiO2 ratio (mean, sd)	318.2	180.0	314.7	165.0	326.6	212.9	6.3
	SAPS II score (mean, sd)	46.6	10.9	46.2	10.0	47.6	12.9	11.6
Common	Diabetes (n, %)	5.1	2.0	3.1	1.8	2.0	2.7	6.4
	Evacuation of cerebral hematoma or lobectomy (n, %)	51.6	20.5	37.6	21.1	14.0	18.9	5.5
	Decompressive craniectomy (n, %)	38.7	15.4	26.7	15.0	12.0	16.2	3.3
	Pneumonia before increased ICP (n, %)	42.7	16.9	29.7	16.7	13.0	17.6	2.4
	Nosological entity: Severe trauma (n, %)	57.2	38.4	28.2	37.6	29.0	39.2	3.3
	Arterial pH (mean, sd)	7.4	0.1	7.4	0.1	7.3	0.1	9.4
	PaO2/FiO2 ratio (mean, sd)	319.3	185.0	316.3	172.8	326.6	212.9	5.4
	SAPS II score (mean, sd)	48.2	10.8	48.4	9.9	47.6	12.9	7.4
Entire	Female patients (n, %)	113.6	45.1	82.6	46.4	31.0	41.9	9.1
	Diabetes (n, %)	8.0	3.2	6.0	3.4	2.0	2.7	3.8
	Evacuation of subdural or extradural hematoma (n, %)	23.5	9.3	15.5	8.7	8.0	10.8	7.1
	External ventricular drain (n, %)	104.0	41.3	79.0	44.4	25.0	33.8	21.9
	Evacuation of cerebral hematoma or lobectomy (n, %)	44.3	17.6	30.3	17.0	14.0	18.9	5.0
	Decompressive craniectomy (n, %)	46.4	18.4	34.4	19.3	12.0	16.2	8.2
	Blood transfusion before admission (n, %)	26.5	10.5	17.5	9.8	9.0	12.2	7.5
	Osmotherapy (n, %)	109.6	43.5	72.6	40.8	37.0	50.0	18.6
	Pneumonia before increased ICP (n, %)	45.5	18.1	32.5	18.3	13.0	17.6	1.8
	Nosological entity: Severe trauma (n, %)	77.1	30.6	48.1	27.0	29.0	39.2	26.0
	Haemoglobin, g/dL (mean, sd)	12.5	2.4	12.7	2.3	12.1	2.5	23.8
	Platelets, counts/mm^3 (mean, sd)	218.5	89.8	224.1	95.5	205.1	74.2	22.3
	Serum creatinine, mmol/L (mean, sd)	69.7	30.0	69.1	28.6	71.1	33.3	6.4
	Arterial pH (mean, sd)	7.3	0.1	7.3	0.1	7.3	0.1	4.7
	Serum proteins, g/L (mean, sd)	59.4	8.9	59.4	8.6	59.6	9.7	3.1
	PaO2/FiO2 ratio (mean, sd)	308.1	187.3	300.3	175.9	326.6	212.9	13.5
	SAPS II score (mean, sd)	48.3	11.1	48.7	10.2	47.6	12.9	9.7

The PS-adjusted samples for matched analysis (FM) of dichotomised Glasgow Outcome Scale score at 3 months according to the covariate sets. Qualitative characteristics are presented by using the matched effective (n) and the matched percentage. Continuous characteristics are presented with weighted mean following by matched standard deviation (sd). STD: Standardised differences in %, SAPS: Simplified Acute Physiology Score, ICP: Intracranial Pressure, PaO2: arterial partial Pressure of Oxygen, and FiO2: Fraction of Inspired Oxygen.
